# Direct biomechanical manipulation of human gait stability: A systematic review

**DOI:** 10.1371/journal.pone.0305564

**Published:** 2024-07-11

**Authors:** Bram Sterke, Saher Jabeen, Patricia Baines, Heike Vallery, Gerard Ribbers, Majanka Heijenbrok-Kal

**Affiliations:** 1 Rehabilitation Medicine, Erasmus Medical Center, Rotterdam, The Netherlands; 2 Department of Biomechanical Engineering, Technical University of Delft, Delft, The Netherlands; 3 Rijndam Rehabilitation Center, Rotterdam, The Netherlands; University rehabilitation institute, SLOVENIA

## Abstract

People fall more often when their gait stability is reduced. Gait stability can be directly manipulated by exerting forces or moments onto a person, ranging from simple walking sticks to complex wearable robotics. A systematic review of the literature was performed to determine: What is the level of evidence for different types of mechanical manipulations on improving gait stability? The study was registered at PROSPERO (CRD42020180631). Databases Embase, Medline All, Web of Science Core Collection, Cochrane Central Register of Controlled Trials, and Google Scholar were searched. The final search was conducted on the 1st of December, 2022. The included studies contained mechanical devices that influence gait stability for both impaired and non-impaired subjects. Studies performed with prosthetic devices, passive orthoses, and analysing post-training effects were excluded. An adapted NIH quality assessment tool was used to assess the study quality and risk of bias. Studies were grouped based on the type of device, point of application, and direction of forces and moments. For each device type, a best-evidence synthesis was performed to quantify the level of evidence based on the type of validity of the reported outcome measures and the study quality assessment score. Impaired and non-impaired study participants were considered separately. From a total of 4701 papers, 53 were included in our analysis. For impaired subjects, indicative evidence was found for medio-lateral pelvis stabilisation for improving gait stability, while limited evidence was found for hip joint assistance and canes. For non-impaired subjects, moderate evidence was found for medio-lateral pelvis stabilisation and limited evidence for body weight support. For all other device types, either indicative or insufficient evidence was found for improving gait stability. Our findings also highlight the lack of consensus on outcome measures amongst studies of devices focused on manipulating gait.

## Introduction

Falling is a major cause of morbidity and mortality in our society [1]. Falls often occur during gait [2], possibly caused by poor balance [3]. A decline in balance during gait, with increased gait variability and an associated increased risk of falls, correlates with aging [4] and with the presence of vestibular, cerebellar, functional, or other neurological diseases [5]. When standing, balance can, for example, be defined as an individual’s ability to maintain their center of mass (CoM) within a base of support (BoS) [3, 5–7] or as “the continuous and adequate adaptation of body posture to avoid falling” [8]. When walking, it is suggested that the vertical projection of the CoM plus its velocity times a factor should be within the BoS [7]. When describing balance during walking, i.e. gait, ‘gait stability’ is an important indicator [5].

Gait stability is a broad concept that can generally be defined as the ability to keep walking regardless of disturbances or the presence of control errors [9, 10]. A multitude of definitions for the stability of bipedal gait exist. Some frequently used ones are summarized in Bioinspired Legged Locomotion, Chapter 4.1 [11]. Kuo and Donelan (2010) distinguish local stability, i.e. step-to-step stability concerned with small deviations from nominal gait, and global stability, i.e. a person’s susceptibility to falling [12]. Although the global definition has more clinical relevance, it is proposed that local stability is a useful indicator of walking balance [3, 5, 12].

Gait stability can be assessed with a wide variety of measures, each with its own level and type of validity [3, 13, 14]. For example, kinematic variability measures show convergent validity in experimental studies, which reflects an experimentally induced change in stability [3]. For the more clinical spatio-temporal measures, convergent validity is often not reported, but predictive validity in observational studies does exist [13], which describes the correlation between the measure and probability of falling. Lab-based stability measures, such as Lyapunov exponents, usually require kinematic and/or kinetic data obtained during walking, with post-processing, for their calculation [13, 15]. Clinical measures are often based on discrete score assignments or simple units of measure [13]. Consequently, they provide an indirect evaluation of balance and gait stability. For the purpose of keeping this review and search more inclusive, both lab-based and clinical measures of gait stability are considered.

Modulating gait stability can either be done directly, by (mechanical) manipulation [16], or indirectly, through training [17]. Mechanical manipulation is here defined as the transmission of forces, or moments, onto a body part, intending to influence human body kinematics. These include external forces and moments applied with respect to an external reference frame (e.g., the floor or wall) or internal forces and moments applied to parts of the body by means of a (wearable) power source (e.g., an actuator).

The complexity of gait stability manipulation and assessment and the desire to improve rehabilitation has encouraged the development of robotic tools [8] that can either improve or disrupt balance to train balance [18, 19]. However, which body part must be manipulated for maximum effect remains a challenge and depends on the user’s limitations. While understanding of mechanisms underlying gait stability is improving [13, 20, 21], limited evidence exists on how various assistive devices perform in terms of gait stability.

When looking at balance, it has been reported that even small, haptic, or vibrotactile, forces can improve balance performance when the point of application is chosen correctly, such as on the hand [22] or on the hip [23]. It has also been shown that the point of application of a force on the upper body greatly influences gait velocity [24]. Similarly, the sensitivity of human gait stability to external forces or moments might vary between their points of application on the body. When developing novel devices, such as cold-gas thrusters, which can generate a linear force impulse [25], choosing the optimal point of application and impulse direction might be pivotal.

Various reviews exist on the definitions of gait stability [3, 13, 14] and on how devices are used to assess balance [8, 26]. To our knowledge, no overview exists on how actuated devices influence human gait stability and where and how they apply forces and moments on the body.

The primary aim of the study is to answer the following question: What is the level of evidence for different types of mechanical manipulations on improving gait stability? For this, we divided device type based on the point of application of forces and moments and their directions. The secondary aim is to create an overview of devices that directly impact gait stability and their manipulation characteristics, such as point of application of forces, force direction, control strategy, and peak force magnitude.

Furthermore, we categorise the reported outcome measures and their types of validity. Due to the heterogeneity of the reported outcome measures, this review will employ a best-evidence synthesis (BES) in order to objectify findings and compare amongst studies [27].

## Methods

### Study protocol and search strategy

The study protocol of this systematic review is registered on PROSPERO (CRD42020180631). The search strategy was developed according to the method described by Bramer et al. (2017) [28] and executed in collaboration with an information specialist from the Erasmus MC Medical Library, Rotterdam. In total, five databases were searched: Embase (Embase.com), Medline All (Ovid), Web of Science Core Collection (Web of Knowledge), Cochrane Central Register of Controlled Trials (Wiley), and Google Scholar. The databases were searched from inception to the 1st of December, 2022. The PRISMA guidelines were used for screening and reporting [29].

In summary, the search strategy was constructed by joining the following—synonyms and antonyms of—three concepts by AND; 1) gait/balance stability/symmetry; 2) (bio-)mechanical/kinematic manipulation; 3) devices/aids/robots. In Embase and Medline the index terms ‘gait’ and ‘walking’ were used to better define the scope. For redundancy, specific outcome measures keywords, such as ‘Lyapunov’, were joined to concept 3) with OR. The full search strategy for all databases can be found in the S1 Appendix.

Two authors (B.S. and S.J.) independently screened titles and abstracts of all identified studies and subsequently reviewed a selection of full texts based on the in- and exclusion criteria described in the next section. Any disagreements were arbitrated by P.B. and M.H.

### Inclusion and exclusion criteria

To be included, studies had to: 1- contain any form of (mechanical) manipulation of gait stability in non-impaired subjects or in individuals with a locomotor problem due to a neurological or orthopedic condition, 2- have a pre-post intervention study design, 3- be written in English, and 4- be performed on humans. All peer-reviewed published studies were included, with no limit on the year of publication.

Studies were excluded if they: 1- contained a training phase between baseline and outcome measurements, 2- were performed in water, 3- investigated prosthetic devices, passive orthoses (kinetic tapes, elastic bands, rigid links locking joints, insoles), surgical procedures, functional electrical stimulation, visual feedback, vestibular sensory manipulation, vibrational feedback, exoskeletons with a fully-enforced multi-limb kinematic trajectory, 4- were performed in children aged less than 16, or 5- contained five or less participants (pilots and case reports), and 6- studies with perturbations were excluded unless the perturbation was applied to increase the challenge for the subject, while the main aim of the study was investigating a stabilising force or intervention. As literature that primarily addresses our research question is sparse, papers that look at gait stability in passing were included, as long as they enabled drawing conclusions on our research question.

In our initial PROSPERO registration, dead mass and elastic bands were also included. These were later dropped to focus the study on devices that provide external forces and moments or internal forces generated by a wearable actuator.

### Data collection

For the included studies, data was collected by one of the researchers and reviewed by the other researcher. The following information was collected: 1- intervention and device type, 2- point of application of forces on the body, 3- force characteristics (i.e. main force direction, control strategy, and peak force magnitude), 4- population description (i.e. sample size, impairment, age mean and standard deviation), 5- intervention protocol, 6- reported outcome measures, and 7- main findings of the study).

### Data analysis

Articles were grouped based on the point of application of forces and moments, and their directions. The evidence for impaired and non-impaired subjects was analysed separately. Instead of the meta-analysis mentioned in the preregistered protocol, a best-evidence synthesis (BES) was performed to allow comparison across heterogeneity outcome measures. The BES was based on; 1- the type of validity of the reported outcome measures and 2- the study’s quality and risk of bias score. Details of both criteria are described below. If a paper described multiple patient populations or multiple interventions, these were considered as separate studies if data presentation allowed separate interpretation. If not, the study was excluded. If a paper described multiple settings of one intervention type only the setting that showed the largest impact on gait stability was used.

#### Outcome measure validity

Multiple reviews describe the various types of validity outcome measures [3, 13, 14, 30]. For the current review we used the categorisation and annotated type of validity as mentioned by Bruijn et al. (2013) [3], updated with recent literature [4, 15, 20, 31–40]. The system distinguishes four types of validity; 1) *Construct validity*—Whether the existence of a relationship between a measure and the probability of falling is plausible [3]. 2) *Predictive validity in models*—Whether the measure predicts a probability of falling in a simple model of human gait [41]. 3) *Convergent validity in experimental studies*—Whether the measure reflects an experimentally induced change in stability [42]. 4) *Predictive validity in observational studies*—Whether there is a correlation between the measure and the incidence—or probability—of falling in observational studies [43].

The categorisation and evidence for the various validity types can be found in Table 1. For the purpose of this study, we selected outcome measures for our analysis if at least predictive validity in observational studies was found. In the preregistration, we mentioned a broad range of outcome measures, e.g. maximum Lyapunov exponent, maximum Floquet multiplier, variability measures, long-range correlations, etc.. Upon occurrence in the included articles more measures were added to the inventory in Table 1. Outcome measures that were not mentioned in the included articles are not described in this overview.

**Table 1:** Types of validity for outcome measures of gait stability. The references are provided for *construct* validity, predictive validity in *models*, *convergent* validity, and predictive validity in *observational* studies. F denotes evidence for falsification of support for a certain type of validity,—denotes no support or falsification of support for a certain type of validity was found.

Category	Measures	Types of validity			
		Construct	Models	Convergent	Observational
Lyapunov exponents	λ	[3, 44]	[3]	[3, 15, 45]	[3, 15]
Kinematic variability	*σ*_SW_, $\sigma_{t_{\text{stride}}}$, etc.	[31]	[3]	[3, 13]	[3, 14]
Angular momentum	*H*_r_	[13, 46]	[46–48]	[13, 32]	[13, 33]
Orbital stability	FM	[3, 34]	[3]	F [3, 35]	[3, 36]
Extrapolated CoM	XCoM, MoS	[3, 49]	[3, 49]	F [3, 32, 37, 38]	[13, 20]
Velocity	WS	-	-	F [30, 50]	[14, 30]
Symmetry	*t*_stance_△, DST△	-	-	F [30]	[30, 39, 40]
Kinematics	SW, SL, *l*_stride_	-	-	F [3, 51]	[3, 4, 14]
Temporal	*t*_step_, *t*_stride_, etc.	-	-	-	[52]
Ratio	*t*_stance_-ratio, etc.	-	-	-	-
Correlations	SW v. Pelvis ROM, etc.	-	-	-	-

#### Quality and risk of bias score

In order to assess each study’s quality and risk of bias, we adopted the “Quality Assessment Tool for Before-After (Pre-Post) Studies With No Control Group” published by the National Institutes of Health (NIH) [53]. Two authors (B.S. and S.J.) scored each article independently, and after discussion, a final study score was awarded. Any conflicts were resolved by P.B. and M.H.. Some questions were deemed more important to the goal of our study, therefore their score weights were increased, specifically questions: 3 (intended user group), 5 (sample size), 10 (statistical analysis), and 11 (multiple base-line measurements). Questions 4 (participant enrollment) and 8 (blinding) were deemed Non-Applicable (NA) as these questions are irrelevant for our comparison. Our final questionnaire is provided in the S1 Table. Quality scores of 11 or higher were classified as ‘excellent’ quality, 8 to 10 were classified as ‘sufficient,’ and 7 or lower were ‘poor’ quality.

#### Best-evidence synthesis

Based on a BES, the studies are categorised into five levels of evidence, ranging from Strong to Insufficient. The categorisation is based on the quality score and outcome measures’ type of validity. The full description is provided in Table 2. As the current study focuses on the direct effect of manipulations on gait stability during experiments, convergent validity was deemed most relevant. For the purpose of counting conflicting evidence, studies reporting a positive impact on gait stability were awarded a +1, studies reporting a negative impact received a -1, and those without conclusive findings received a zero. The net sum of these points determined the level of evidence in Table 2. For example, when four studies report an improvement in gait stability (+4) through measures with convergent validity, and one study reported a reduction in gait stability (-1) with similar outcome measure validity, the overall BES conclusion for the device based on these studies (+3) would be deemed ‘Strong’.

**Table 2:** Best-evidence synthesis (BES) ranking definition. The outcome of the BES can range from strong to insufficient evidence.

Strong	Provided by statistically significant findings in ≥ 3 papers with quality score ≥ 11 and measures with at least convergent validity
Moderate	Provided by statistically significant findings in 2 paper with quality score ≥ 11 and measures with at least convergent validity
Limited	Provided by statistically significant findings in 1 papers with quality score ≥ 8 and measures with construct and observational validity AND 2 papers with quality score ≥ 8 and measures with at least observational validity
Indicative	Provided by statistically significant findings in 1 papers with quality score ≥ 8 and measures with construct and observational validity OR 2 papers with quality score ≥ 8 and measures with at least observational validity
Insufficient	In the case that none of the above-mentioned criteria are met OR In the case of conflicting results among studies OR In the case of no eligible studies

## Results

### Search results and identification of studies

**Fig 1:**
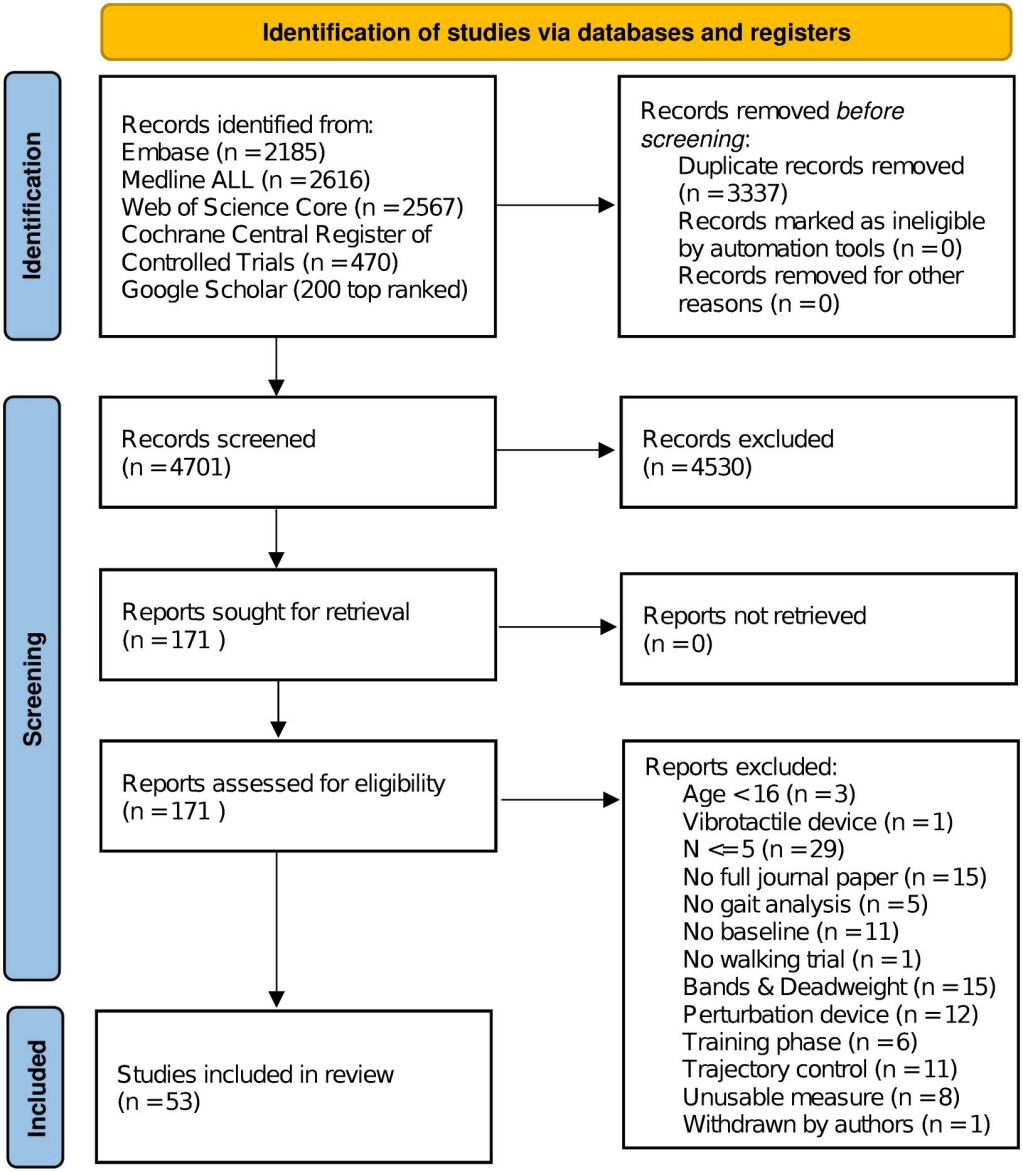
PRISMA flowchart showing the literature screening process.

All searches combined resulted in 4701 articles for screening. Title and abstract screening of all articles resulted in 171 articles for full-text screening. Assessment for eligibility led to the inclusion of 53 papers for analysis, see Fig 1 for more details.

Preregistration of protocols and outcome measures was present in three studies [54–56]. Original data was made publicly available in seven studies [16, 54, 56–60], whereas seven studies mentioned data to be readily available upon request [61–67].

### Categorisation

Overall 17 papers focused solely on impaired subjects, 28 papers focused on non-impaired subjects, and seven papers looked at both. One study added an artificial impairment to non-impaired participants by limiting movement of the leg [68]. A total of 81 interventions, taken from 53 articles, were included in the BES. A full description of all studies and the device characteristics can be found in Table 3.

**Table 3:** Descriptions of study and device characteristics. VT = Vertical, AP = anterior-posterior, ML = medio-lateral, NR = not reported, ABI = acquired brain injury, SCI = spinal cord injury, BWS = body weight support, GRF = ground reaction forces, PD = Parkinson’s disease, HD = Huntington’s disease, NR = not reported, BW = body weight.

Index	Point of application	Device description	Controller	Force characteristics *Primary direction*	*Magnitude*	Impairment	Population	
							Age (SD)	N
Zoffili et al. [92]	Hands	Walking pole (for hiking)	Self-induced GRF	VT	NR	Healthy	30.9 (8.2)	21
Zhang et al. [83]	Hands	Conventional rolling walker	Self-induced GRF	VT	NR	PD	66 (median)	6
		Self-navigating motorized walker.	Self-induced GRF + autonomous forward pull	VT + AP	NR	PD	66 (median)	6
Wan et al. [86]	Hands	Motorized walker	Self-induced GRF + autonomous forward pull	VT + AP	NR	Healthy	28.57 (3.3)	11
		Rollator	Self-induced GRF	VT	NR	Healthy	28.57 (3.3)	11
Sonntag et al. [88]	Hands	Forearm crutch	Self-induced GRF	VT	NR	Hip Arthroplasty	68.4 (NR)	19
Sorrento et al. [64]	Hands	Instrumented cane	Self-induced GRF	VT	NR	A1. Stroke (high functioning)	70.9 (2.9)	7
		Instrumented cane	Self-induced GRF	VT	NR	A2. Stroke (low functioning)	70.4 (3.2)	7
		Instrumented cane	Self-induced GRF	VT	NR	B1. Healthy	71.8 (2.7)	14
		Robot-generated haptic leash forces	Autonomous forward pull	AP	15N	A1. Stroke (high functioning)	70.9 (2.9)	7
		Robot-generated haptic leash forces	Autonomous forward pull	AP	15N	A2. Stroke (low functioning)	70.4 (3.2)	7
		Robot-generated haptic leash forces	Autonomous forward pull	AP	15N	B1. Healthy	71.8 (2.7)	14
Seiferheld et al. [85]	Hands	Conventional rolling walker	Self-induced GRF	VT	NR	Healthy	24.5 (3.1)	18
		Walker with movable vertical handlebars (Crosswalker, Human Walking ApS)	Self-induced GRF	VT	NR	Healthy	24.5 (3.1)	18
Polese et al. [89]	Hands	Walking sticks (canes or crutches)	Self-induced GRF	VT	NR	Stroke Chronic	56.5 (7.4)	19
Maguire et al. [90]	Hands	Cane	Self-induced GRF	VT	NR	Stroke Sub-acute	64 (14)	13
Kloos et al. [84]	Hands	Cane	Self-induced GRF	VT	NR	HD	49.3 (11)	21
		Conventional non-rolling walker	Self-induced GRF	VT	NR	HD	49.3 (11)	21
		Two-wheeled walker	Self-induced GRF	VT	NR	HD	49.3 (11)	21
		Three-wheeled walker	Self-induced GRF	VT	NR	HD	49.3 (11)	21
		Four-wheeled walker	Self-induced GRF	VT	NR	HD	49.3 (11)	21
Jayakaran et al. [91]	Hands	Cane for balance and support	Self-induced GRF	VT	NR	Healthy	44.74 (10)	27
Ijmker et al. [87]	Hands	Cane	Self-induced GRF	VT	NR	A1. Stroke (dependent)	57.1 (13)	12
		Cane	Self-induced GRF	VT	NR	A2. Stroke (independent)	46.9 (17.3)	12
		Handrail	Self-induced support force	VT	NR	A1. Stroke (dependent)	57.1 (13)	12
		Handrail	Self-induced support force	VT	NR	A2. Stroke (independent)	46.9 (17.3)	12
Bannwart et al. [61]	Trunk	3D BWS (FLOAT, Reha-Stim Medtec AG)	Autonomous	VT	30% BW	Healthy	26.8 (3.5)	21
		3D BWS (FLOAT, Reha-Stim Medtec AG)	Autonomous	VT + ML	30% BW + 120 Ns/m ML damping	Healthy	26.8 (3.5)	21
Best et al. [72]	Trunk	Inverted pendulum with dead mass oscillation IN and OUT of phase in a backpack	Passive spring	ML	NR	Healthy	21.8 (1)	12
Clark et al. [99]	Trunk	2D BWS (LiteGait)	Passive spring	VT	30% BW	ABI	38.7 (15.3)	17
	Trunk	2D BWS (LiteGait) plus handrail	Passive spring + Self-induced support force	VT	30% BW + NR	ABI	38.7 (15.3)	17
Dragunas et al. [70]	Trunk	Motorized 2D BWS (Aretech, Ashburn, AV)	Autonomous	VT + ML	20–60% BW	Healthy	26 (4)	8
Goncalves et al. [69]	Trunk	BWS	Passive linear spring	VT	30% BWS	Healthy	22.25 (3.02)	8
Ignasiak et al. [59]	Trunk	BWS	Not reported	VT	20–40% BW	Healthy	27 (4.2)	20
Lemus et al. [57]	Trunk	Control moment gyroscopes in backpack (GyBAR)	Virtual rotational damper and spring-damper	AP	100 Nm/rad, 30 Nms/rad	Healthy	35 (NR)	10
Pillar et al. [71]	Trunk	BWS	Passive spring-damper	VT	20% BW	Healthy	25–50 (NR)	6
		BWS	Passive spring-damper	VT	20% BW	Hemiplegia	40–84 (NR)	18
Qian et al. [68]	Pelvis and Thigh	Hip exoskeleton	Autonomous	ML axis	19.8 Nm	Artificial impairment	23.4 (2.6)	7
Livolsi et al. [66]	Pelvis and Thigh	Active Pelvis Orthosis (APO)	Autonomous	ML axis	22 Nm max	Stroke-knee HE	49 (10.6)	6
Livolsi et al. [66]	Pelvis and Thigh	Active Pelvis Orthosis (APO)	Autonomous	ML axis	23 Nm max	Stroke	53.38 (11.5)	8
Lee et al. [54]	Pelvis and thigh	Hip-exoskeleton applying joint torque to hip (GEMS, Samsung)	Autonomous	ML axis	3.13–9.70 Nm	Elderly	74.1 (4.18)	30
Monaco et al. [63]	Pelvis and thigh	Hip-exoskeleton applying joint torque to hip (Custom, APO)	Autonomous	ML axis	Max 14 Nm	Elderly	68.9 (5.1)	8
Park et al. [96]	Pelvis and thigh	Hip-exoskeleton applying joint torque to hip (backX AC, US Bionics Inc., Berkeley, CA)	Autonomous	ML axis	NR	Healthy	M = 24.8 (4.2), F = 24.1(1.9)	20
Hsu et al. [55]	Shank and Foot	Ankle Foot Orthosis with dynamic control (Custom, IT-AFO) applying joint torque	Passive spring-damper	ML axis	0.625 kgf + 1 Ns/m	Stroke	51.14 (18.5)	7
Norris et al. [98]	Shank and Foot	Powered AFO applying ankle torque	Autonomous	ML axis	Max 160 N	Healthy	23.3 (1.6)	9
Heitkamp et al. [100]	Shank	Lateral force field applied to the shank	Simulated linear spring	ML	180 N/m	Healthy	24 (2)	12
Nyberg et al. [101]	Shank	Medial and Lateral force field applied to the shank	Simulated force-field	ML	1150–3500 N/m	Healthy	24 (3)	11
Reimold et al. [102]	Shank	Lateral force field applied to the shank	Simulated force-field	ML	Effective ML stiffness 180 N/m	Stroke	60 (17)	10
Yen et al. [103]	Shank	AP forward force applied to the shank	Autonomous	AP	21.1 (5.0) N	Stroke	61.5 (6)	9
Bacek et al. [95]	Thigh and shank	Unilateral knee assistive device	Autonomous	ML axis	0.05 Nm/kg BW	Healthy	32 (7)	7
Haufe et al. [94]	Thigh and shank	Knee assistive device	Autonomous	ML axis	212 N	Healthy	27(NR)	8
Chinimilli et al. [93]	Thigh and shank	Knee assistive device applying supportive joint torque	Autonomous	ML axis	11.26 Nm	Healthy	24.3 (2.9)	11
Choi et al. [67]	Shank and foot	Powered AFO (pneumatic)	Autonomous	AP, ML axes	NR	Healthy	30 (4)	7
Galle et al. [97]	Shank and foot	Powered AFO	Autonomous	ML axis	0.11(±0.2) W/kg per leg	Elderly	69.3 (3.5)	7
Vashista et al. [104]	Pelvis	Tethered pelvis assist device	Passive spring	AP, ML, VT	4.04 kN/m	Healthy	27 (2.33)	8
Bruijn et al. [58]	Pelvis	Medio-lateral pelvis forces	Passive spring-damper	ML	~2000 N/m	Healthy	31.4 (6.6)	10
van Leeuwen et al. [79]	Pelvis	Medio-lateral pelvis forces	Passive spring	ML	1260 N/m	Healthy	NR (NR)	20
Dean et al. [73]	Pelvis	Medio-lateral pelvis forces	Passive spring-damper	ML	1200 N/m, 20 Ns/m	Healthy	25.4 (3.6)	8
		Medio-lateral pelvis forces	Passive spring-damper	ML	1200 N/m, 20 N.s/m	Elderly	73.4 (4.2)	10
Donelan et al. [74]	Pelvis	Medio-lateral pelvis forces	Passive spring-damper	ML	1700 N/m, 14 Ns/m	Healthy	NR (NR)	10
Dragunas et al. [80]	Pelvis	Medio-lateral force applied to pelvis by series-elastic actuator (Agility Trainer)	Simulated damper	ML	50 Ns/m	Stroke	59 (7)	9
		Medio-lateral force applied to pelvis by series-elastic actuator (Agility Trainer)	Simulated damper	ML	50 Ns/m	Healthy	61 (6)	9
Frame et al. [75]	Pelvis	Medio-lateral pelvis forces	Simulated spring	ML	1260 N/m	Stroke	50 (16)	13
		Medio-lateral pelvis forces	Simulated spring	ML	1260 N/m	Healthy	49 (16)	18
Goncalves et al. [69]	Pelvis	BWS applied to the pelvis by bicycle seat	Passive linear spring	VT (AP, ML restricted)	30% BW	Healthy	22.25 (3.02)	8
Graham et al. [105]	Pelvis	BWS (KineAssist)	Autonomous	Pelvis sway, roll, yaw locked	80% BW, pelvis NR	Healthy	26.8 (4.9)	20
	Pelvis and Trunk	BWS (KineAssist) + Trunk frame	Autonomous	Pelvis and trunk sway, roll, yaw locked.	80% BW, pelvis NR	Healthy	26.8 (4.9)	20
Ijmker et al. [76]	Pelvis	Medio-lateral pelvis forces	Passive spring-damper	ML	760–1820 N/m, 15.9–32 Ns/m	Healthy	20 (1.2)	14
Koopman et al. [77]	Pelvis	Medio-lateral pelvis forces	Virtual spring	ML	0–4500 N/m	Healthy	28.8 (3.9)	6
LinJ et al. [82]	Pelvis	Medio-lateral pelvis forces	Autonomous	ML	max 8 to 12% BW	iSCI	51.5 (12.7)	16
LinJ et al. [62]	Pelvis	Medio-lateral pelvis forces	Autonomous (Abrupt, gradual, varied forces)	ML	max 8 to 12% BW	iSCI	48.2 (12.28)	12
Mahaki et al. [56]	Pelvis	Medio-lateral pelvis forces	Passive spring	ML	1260 N/m	Healthy	27.7 (4.78)	10
Mahaki et al. [16]	Pelvis	Medio-lateral pelvic forces—free or restricted in transversal and/or frontal plane	Passive spring	ML	1260 N/m	Healthy	27.5 (2.4)	11
Matsubara et al. [81]	Pelvis	Medio-lateral pelvis forces	Passive spring-damper	ML	1027 N/m, 2.3 Ns/m	iSCI	56.2 (9.6)	9
Ochs et al. [60]	Pelvis	Medio-lateral pelvis forces generated by series-elastic actuator (Agility Trainer)	Simulated damper	ML	40 Ns/m, max 80 N	iSCI	49.5 (15.5)	11
		Medio-lateral pelvis forces generated by series-elastic actuator (Agility Trainer)	Simulated damper	ML	40 Ns/m, max 80 N	Healthy	47.3 (16.1)	12
Wu et al. [78]	Pelvis	Medio-lateral pelvis forces generated by series-elastic actuator (Agility Trainer)	Simulated damper	ML	427 ± 78 Ns/m	Healthy	24 (4)	10
		Medio-lateral force applied to hip generated by series-elastic actuator (Agility Trainer)	Simulated damper	ML	427 ± 78 Ns/m	iSCI	58 (8)	7
Walker et al. [65]	Pelvis	Lateral force applied to pelvis/trunk by series elastic actuator (Custom)	Autonomous	ML	2.5–5% BW, max 100 N	Healthy	NR (NR)	10
	Pelvis and Hand	Lateral force applied to pelvis/trunk and handrail	Autonomous + self-induced support force	ML	2.5–5% BW, max 100 N	Healthy	NR (NR)	10

Studies were divided into four groups; *Trunk* (n = 8), *Pelvis* (n = 19), *Upper Extremity* (n = 11), and *Lower Extremity* (n = 16), where Goncalves et al. [69] is counted towards both the *Trunk* and the *Pelvis* group for their two device types.

**Fig 2:**
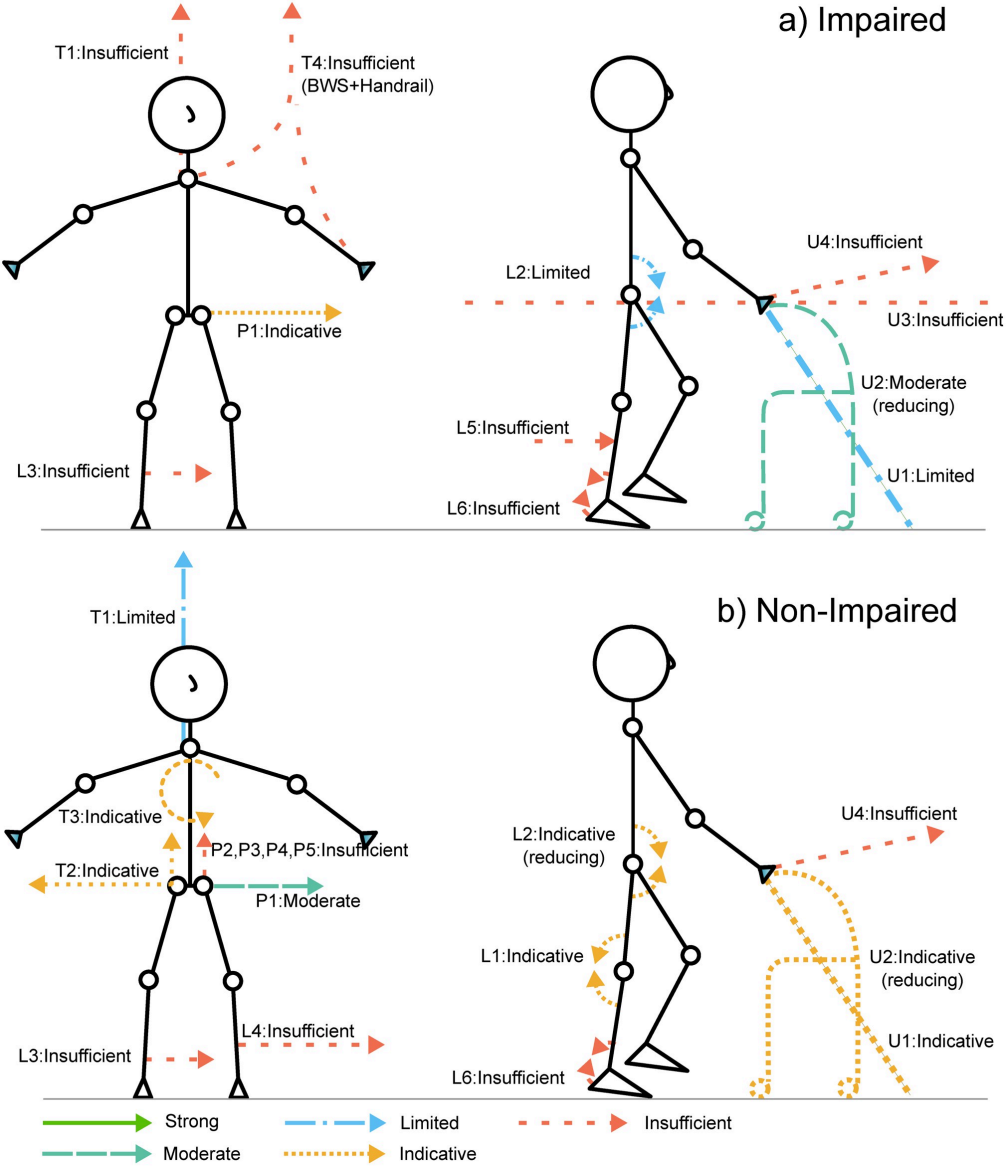
Best-evidence synthesis (BES) conclusions for a) impaired and b) non-impaired subjects. Arrows indicate the point of application and main direction(s) of the forces and moments. Colours and line type indicate the level of evidence. The letter and number combinations are indices for the device types. P = Pelvis, P1: Medio-lateral forces, P2: 3D forces, P3: Vertical body weight support (BWS) unloading, while pelvis’ sway, roll, and yaw motions locked, P4: Vertical BWS unloading force, with trunk motion, pelvis’ sway, roll, and yaw motions locked, P5: Medio-lateral pelvis manipulation combined with handrail. L = Lower extremity, L1: Knee joint torque applied over thigh and shank, L2: Hip joint torque applied to pelvis and thigh, L3: Medial damping force applied to the shank, L4: Lateral damping force applied to the shank, L5: Forward assistance force applied to the shank, L6: Ankle joint torque applied by a powered ankle foot orthosis. T = Trunk, T1: Vertical BWS to the trunk, T2: Vertical BWS plus medio-lateral damping, T3: Flywheel-based torques applied to the trunk. Manipulation of the frontal plane medio-lateral trunk angle, T4: BWS plus handrail. U = Upper extremity, U1: Walking poles, walking sticks, cane, and crutches, U2: Walkers, ranging from non-wheeled to four-wheeled, U3: Handrail, U4: Forward force provided to the hand by a leash.

For the BES we identified 27 different subgroups based on population and type of intervention. A full description of each subgroup can be found in Table 4. A visual representation of the evidence levels concluded during the BES can be found in Fig 2. The outcomes of the quality and risk of bias assessment can be found in the S2 Table.

**Table 4:** Best-evidence synthesis (BES) conclusions table. For each device and intervention type, the overall BES is presented and followed by a description of the findings of the articles that were considered within that group. The change of the outcome measure as a result of using the device is denoted as; ↓—decrease, ↑—increase, ~—negligible, *—significant, NR—not reported.

Device and intervention	BES Conclusion	Ref.	Main findings	Quality & Bias	Outcomes	Weight
P1: Medio-lateral forces applied to the pelvis, typically a physical or simulated spring, damper, or a combination thereof.	Moderate evidence for improving gait stability in non-impaired subjects	[58]	Convergent: Local dynamic stability significantly improved.	9	SW↓*, λ of ML trunk velocity↓*, *t*_stride_~, ML trunk RoM ↓*	+1
		[73]	Observational: Step width reduced significantly.	11	SW↓*, *σ*_SW_↓, SL~, *σ*_SL_↑	+1
		[74]	Convergent: Step width variability reduced significantly.	10	SW↓*, *σ*_SW_↓*, SL~, *σ*_SL_↓, *d*_CoM,ML_↓*	+1
		[80]	No significant finding reported.	13	SW~, *σ*_SW_~, CoM state and foot placement correlation (R^2^)~	0
		[75]	Observational: Step width and ML-CoM sway significantly reduced.	11	SW↓*, *σ*_SW_~, pelvis ML sway↓*, trunk ML sway↓*, var *σ* ML foot placement↓*, ML foot placement ratio↑*, *H*_r_↑*	+1
		[76]	Convergent: Step width variability reduced significantly.	12	SW↓*, *σ*_SW_↓*, *l*_stride_~, *σ* of *l*_stride_~,*σ* of AP trunk acceleration~, *σ* of ML trunk acceleration↓*	+1
		[56]	Convergent: Step width variability reduced significantly.	11	SW↓*, *σ*_SW_↓*, foot placement trunk correlation↓*	+1
		[16]	Observational: Step width and step length reduced significantly. Pelvis rotations and displacements in all directions also reduced significantly.	11	SW↓*, SL↓*, *θ* of VT pelvis axis↓*, *θ* of AP pelvis axis↓, *θ* of VT trunk axis ↓*, pelvis ML displacement↓*, pelvis AP displacement↓*, pelvis VT displacement↓*, arm swing↓*	+1
		[60]	Construct and Observational: MoS-min reduce significantly.	11	minimal ML MoS↓*, SW~, *d*_CoM,ML_↓, ML CoM velocity↓	-1
		[77]	Convergent: Step width variability reduced.	6	SW↓, *σ*_SW_ ↓, ML MoS↑, pelvis ML sway↓, *σ* of ML pelvis sway↓	+1
		[65]	Observational: Step width, cadence, and CoM sway reduced significantly.	10	SW↓*, Cadence ↓*, ML CoM sway↓*	+1
		[78]	Observational: Step width and CoM speed reduced significantly.	11	SW↓*, *σ*_SW_~, ML MoS↑, *t*_step_↓, *v*_CoM,ML_↓*, *σ* of ML CoM velocity↓	+1
		[79]	Convergent: Lyapunov exponent significantly reduced.	10	λ 3D CoM↓*, SW↓*	+1
P1: Medio-lateral forces applied to the pelvis, typically a physical or simulated spring, damper, or a combination thereof.	Indicative evidence for improving gait stability in impaired subjects, multiple conflicting reports.	[73]	Observational: Step width reduced significantly.	11	SW↓*, *σ*_SW_↓, SL~, *σ*_SL_↓	+1
		[80]	No significant findings reported.	13	SW~, *σ*_SW_~, CoM state and foot placement correlation (R^2^)~	0
		[75]	Observational: Step width and ML-CoM sway significantly reduced. They excluded any non-responders from the analysis.	11	SW↓*, *σ*_SW_~, pelvis ML sway↓*, trunk ML sway~, *σ* ML foot placement↓*, ML foot placement ratio~, *H*_r_↑*	+1
		[81]	Convergent: Step width variability significantly reduced. Construct and Observational: Minimum MoS in lateral direction significantly reduced.	11	SW↓*, *σ*_SW_↓*, minimal ML MoS↓*, SL~, *σ*_SL_~, XCoM~	-1
		[82]	Observational: Step length and weight shifting on the stronger side significantly increased. Construct and observational: Minimum MoS significantly reduced for the stronger side and reduced non-significantly for the weaker side.	13	SL↑*, min. ML MoS↓*, SST↓, *d*_CoM,ML_↓* (strong side)	0
		[62]	Construct and observational: Minimum MoS significantly reduced for first 30 steps, no significant change during late adaptation.	12	minimal ML MoS↓* (early), minimal ML MoS~(late), ML MoS at heel contact↑, *σ* of minimal ML MoS error^*NR*^, *σ* of ML MoS at heel contact^*NR*^	0
		[60]	No significant findings reported.	11	minimal ML MoS~, SW~, *d*_CoM,ML_↓, *v*_CoM,ML_↓	0
		[78]	Construct and observational: Significant increase in MoS.	11	SW↓*, *σ*_SW_~, ML MoS↑*, *t*_step_↓, *v*_CoM,ML_↓*, *σ* of ML CoM velocity↓	+1
P2: 3D forces applied to the pelvis	Insufficient evidence for non-impaired subjects	[69]	No significant findings reported.	6	ML CoM RoM↓^*NR*^, VT CoM RoM↓^*NR*^, ML shoulder RoM↓^*NR*^, VT shoulder RoM↓^*NR*^, *θ* of ML trunk axis↓^*NR*^	0
		[104]	Observational: Vertical pelvic range of motion decreased significantly.	9	VT pelvis RoM↓*, SST△~, *t*_stance_△~, DST△~, Foot pressure↑*	+1
P3: Vertical body weight support unloading force applied to the lower pelvis. Pelvis’ sway, roll, and yaw motions locked.	Insufficient evidence for non-impaired subjects	[105]	Due to extensive restriction of motion of the body no conclusions on gait stability are drawn.	11	*t*_stance_↑, *t*_stride_~	0
P4: Vertical body weight support unloading force applied to the lower pelvis. Trunk motion locked. Pelvis’ sway, roll, and yaw motions locked.	Insufficient evidence for non-impaired subjects	[105]	Due to extensive restriction of motion of the body no conclusions on gait stability are drawn.	11	*t*_stance_↓*, *t*_stride_~	0
P5: Medio-lateral pelvis manipulation combined with handrail	Insufficient evidence for non-impaired subjects	[65]	Observational: Step width reduced significantly.	10	SW↓*, Cadence↓*, ML CoM sway↓*	+1
L1: Knee joint torque applied over thigh and Shank	Indicative evidence for improving gait stability in non-impaired subjects	[93]	Construct and observational: MoS in AP direction significantly improved.	8	AP MoS↑*, ML MoS↓, λ(left knee angle)↓*, λ(left hip angle)↓*, λ(left ankle angle)↓, λ(right knee angle)↑*, λ(right hip angle)↑*, λ(right ankle angle)↑, MAD(left hip)↓*, MAD(left knee)↓*, MAD(left ankle)↓*, MAD(right hip)↓*, MAD(right knee)↓, MAD(right ankle)↓*, FMmax(left, all joints)↓*, FMmax(right, all joints)↑*, gait symmetry index↓	+1
		[94]	No significant findings reported.	10	*t*_stride_↓*, $\sigma_{t_{\text{stride}}}$~, arm swing △↓*,	0
		[95]	No significant findings reported.	11	Stride height ↓	0
L2: Hip joint torque applied to pelvis and thigh	Indicative evidence for reducing gait stability in non-impaired subjects	[96]	Convergent: λ significantly reduced and kinematic variability significantly increased. Construct and observational: AP MoS significantly reduced and ML MoS significantly increased.	11	SL↓*, SW↑*, GCT↓*, *t*_swing_↑*, DST↓*, minimum foot clearance↓, *σ*_SL_↑*, *σ*_SW_↑*, *σ* of GCT↑*, $\sigma_{t_{\text{swing}}}$↑*, *σ*_DST_↑*, ML MoS↓* (in swing), AP MoS↑* (in swing), ML MoS↑* (at HS), AP MoS↓* (at HS), λ of trunk angular velocity↓*	-1
L2: Hip joint torque applied to pelvis and thigh	Limited evidence for improving gait stability in impaired subjects	[54]	Observational: Gait parameters significantly improved.	9	WS↑*, Cadence↑*, *l*_stride_↑*, SW↑, SST↑*	+1
		[63]	Construct and observational: AP CoM stability and MoS increased significantly.	10	AP CoM stability↑*, AP MoS↑*	+1
		[66]	Stroke with knee hyper extension. No significant findings reported.	11	WS↑*, 6MWT↑	0
		[66]	Stroke without knee hyper extension. No significant findings reported.	11	WS↑*, 6MWT↑	0
		[68]	Observational: Gait symmetry index increased significantly.	11	gait symmetry index↑*	+1
L3: Medial damping force applied to the shank	Insufficient evidence for non-impaired subjects	[100]	Significant correlation between pelvis displacement and step width.	9	ML pelvis displacement and SW correlation*	0
		[101]	No significant findings reported.	9	SW-20↑, SL-20~,	0
L3: Medial damping force applied to the shank	Insufficient evidence for impaired subjects	[102]	No significant findings reported.	12	SW(AL)↑, SL(AL)↓, *r*_FP,ML_(AL)↑, SW(UL)↑, SL(UL)↓, *r*_FP,ML_(UL)↓, ML pelvis displacement and SW correlation(AL,startstep)↑, ML pelvis displacement and SW correlation*(AL,endstep)↓	0
L4: Lateral damping force applied to the shank	Insufficient evidence for non-impaired subjects	[101]	Observational: Step width significantly decreased.	9	SW-10↓*, SL-10~	+1
L5: Forward assistance force applied to the shank	Insufficient evidence for impaired subjects	[103]	Observational: Step length and symmetry significantly increased for affected leg.	11	SL(AL)↑*, SL(UL)↓, SL△↑*, *t*_swing_(AL)~, *t*_swing_(UL)~, *t*_swing_△~	+1
L6: Ankle joint torque applied by a powered ankle foot orthosis	Insufficient evidence for impaired subjects	[55]	Observational: Temporal parameters increased but non-significantly.	10	WS↑, DST↑, *t*_stance_↑	0
		[97]	Observational: Step length increased significantly.	10	SW↓, *σ*_SW_~, SL↑*, *σ*_SL_~	+1
L6: Ankle joint torque applied by a powered ankle foot orthosis	Insufficient evidence for non-impaired subjects	[98]	Construct and observational: FM increased significantly (stability decreased).	8	λ of ankle angle and velocity~, FM of ankle angle↑*	-1
		[67]	Convergent: λ significantly reduced (local stability increased), kinematic variability non-significantly increased.	9	λ of 3D trunk acceleration↓*, *σ* of 3D trunk acceleration↑, ML trunk sway↑*	+1
T1: Vertical body weight support applied via a trunk harness	Limited evidence for improving gait stability in non-impaired subjects	[59]	Convergent: Step width variability significantly decreased.	9	λ of ML CoM position↓, $\sigma_{t_{\text{stride}}}$ ↑, *σ*_SW_↓*	+1
		[70]	Convergent: Step width variability significantly decreased.	8	SW↑*, *σ*_SW_↓*, SL↓, *σ*_SL_↓, ML MoS↓	+1
		[69]	No significant findings reported.	6	ML CoM RoM↓, VT CoM RoM↓, ML shoulder RoM↓, VT shoulder RoM↓, *θ* of ML trunk axis↓	0
		[71]	No significant findings reported.	6	WS-ratio↓, *t*_swing_-ratio~, *t*_stance_-ratio~	0
		[61]	Convergent: Step width variability significantly decreased. Lyapunov exponent significantly decreased (local stability increased).	11	SL↑*, duty cycle↓*, λ of vertical CoM position↓*, λ of vertical CoM velocity↓*, SW↓*, ML CoM sway↓*, ML MoS↓, *σ*_SW_↓*, *σ* of ML CoM sway↓*, λ of ML CoM position↓*, λ of ML CoM velocity↓*	+1
T1: Vertical body weight support applied via a trunk harness	Insufficient evidence for impaired subjects	[71]	No significant findings reported.	6	WS-ratio↑, *t*_swing_-ratio↑, *t*_stance_-ratio↓	0
		[99]	Observational: CoM ML displacement increased significantly.	9	*d*_CoM,ML_ ↑*, *d*_CoM,ML_ phase↓*, *d*_CoM,ML_ frequency↓	-1
T2: Vertical body weight support plus medio-lateral damping	Indicative evidence for improving gait stability in non-impaired subjects	[61]	Convergent: Step width variability significantly decreased. Lyapunov exponent significantly decreased (local stability increased).	11	SL↑, duty cycle↓*, λ of vertical CoM position↓*, λ of vertical CoM velocity↓*, SW↓, ML CoM sway↓*, ML MoS↓, *σ*_SW_↓*, *σ* of ML CoM sway↓*, λ of ML CoM position↓*, λ of ML CoM velocity↓*	+1
T3: Flywheel-based torques applied to the trunk. Manipulation of the frontal plane medio-lateral trunk angle	Indicative evidence for improving gait stability in non-impaired subjects	[72]	Out of phase: Convergent: Lyapunov exponent significantly decreased (local stability increased). construct and observational: MoS significantly increased.	10	SW↓, *σ*_SW_↑, SL↓, *σ*_SL_↑, *t*_step_↓, *σ*_ST_↑ λ of ML CoM velocity↓*, ML MoS↑*, *r*_CoM,ML_↓	+1
		[57]	Observational: Walking distance increased significantly.	8	walking distance↑*, WS~, ML trunk *θ*~, ML angle *θ* velocity~	+1
T4: Body weight support plus handrail	Insufficient evidence for impaired subjects	[99]	Observational: CoM ML displacement phase and frequency decreased significantly.	9	*d*_CoM,ML_↓, *d*_CoM,ML_ phase↓*, *d*_CoM,ML_ frequency↓*	+1
U1: Walking poles, walking sticks, cane, and crutches	Limited evidence for improving gait stability in impaired subjects	[87]	A1. Convergent: Stride time variability reduced significantly. Observational: Stride time increased significantly.	13	*t*_stride_↑*, $\sigma_{t_{\text{stride}}}$↓*, *t*_step_△↑, WS↑	+1
		[87]	A2. Observational: Stride time increased significantly and walking speed reduced significantly.	13	*t*_stride_↑*, $\sigma_{t_{\text{stride}}}$↑, *t*_step_△↑, WS↓*	0
		[88]	Observational: Stance and swing time symmetry, and stride length increased significantly.	10	WS~, *l*_stride_↑*, Cadence↓*, *t*_stance_△↑*, *t*_swing_△ ↑*, DST△↑, *t*_stance_(AL)↑, *t*_stance_(UL)↓, SST↑*, DST↓*	+1
		[89]	Observational: Walking speed increased significantly.	11	WS↑*	+1
		[90]	No significant findings reported.	9	WS↑, SL△↑^*NR*^, SST△↑^*NR*^	0
		[64]	A1. Obervational: Walking speed increased non-significantly. Bilateral Sobolev norm difference increased significantly.	10	WS↑, Sobolev norm↑*	0
		[64]	A2. Observational: Walking speed increased non-significantly. Bilateral Sobolev norm difference increased significantly.	10	WS↑, Sobolev norm↑*	0
		[84]	Cane. Observational: Walking speed and stride length decreased significantly while other gait parameters improved non-significantly.	11	WS↓*, *l*_stride_↓*, *t*_swing_↓, DST↑, BoS↑, *σ*_ST_↑, $\sigma_{l_{\text{stride}}}$↑, $\sigma_{t_{\text{swing}}}$↑, *σ*_DST_↑	0
U1: Walking poles, walking sticks, cane and crutches	Indicative evidence of improving gait stability for non-impaired subjects	[91]	Observational: Temporal parameters improved significantly.	9	Cadence↓, BoS↑, (Ipsilateral: *t*_swing_↓*, *t*_stance_↑*, SST↓, DST↑*) (Contralateral: *t*_swing_~, *t*_stance_~, SST~, DST↑*)	+1
		[64]	B1. Observational: Bilateral Sobolev norm difference increased insignificantly.	10	WS↑, Sobolev norm↑	0
		[92]	Convergent: Local stability in all directions (Lyapunov exponent) improved significantly. Kinematic variability of trunk increased significantly.	9	λ of AP trunk acceleration↓*, λ of ML trunk acceleration↓*, λ of VT trunk acceleration↓*, MAD of ML trunk acceleration↑*, MAD of AP trunk acceleration↑*, MAD of VT trunk acceleration~	+1
U2: Walkers, ranging from non-wheeled to four-wheeled	Moderate evidence of reducing gait stability for impaired subjects	[83]	Four-wheeled walker. Observational: Swing time and step length, respectively, increased and decreased significantly.	10	GCT↑, *t*_swing_↑*, *t*_stance_↑, SL↓*, SH↓, WS↓, gait ratios and symmetries^*NR*^	+1
		[83]	Motorized walker. Observational: Swing time and step length respectively increased and decreased significantly.	10	GCT↑, *t*_swing_↑*, *t*_stance_~, SL↓*, SH↓, WS↓*, gait ratios and symmetries^*NR*^	+1
		[84]	Four-wheeled walker. Observational and construct: BoS decreased significantly.	11	WS↓, *l*_stride_↓, *t*_swing_↑, DST↑, BoS↓*, *σ*_ST_↑, $\sigma_{l_{\text{stride}}}$↑, $\sigma_{t_{\text{swing}}}$↓, *σ*_DST_↑	-1
		[84]	Three-wheeled walker. Convergent: Kinematic variability measures increased significantly.	11	WS↓, *l*_stride_↓, *l*_swing_↓, DST↑*, BoS↓*, *σ*_ST_↑*, $\sigma_{l_{\text{stride}}}$↑, $\sigma_{t_{\text{swing}}}$↑*, *σ*_DST_↑*	-1
		[84]	Two-wheeled walker. Observational: WS, stride length, and BoS decreased significantly.	11	WS↓*, *l*_stride_↓*, *l*_swing_↓, DST↑, BoS↓*, *σ*_ST_↑, $\sigma_{l_{\text{stride}}}$↑, $\sigma_{t_{\text{swing}}}$↑, *σ*_DST_↑*	-1
		[84]	No-wheeled walker. Convergent: Kinematic variability increased significantly.	11	WS↓*, *l*_stride_↓*, *t*_swing_↓, DST↑, BoS↓, *σ*_ST_↑*, $\sigma_{l_{\text{stride}}}$↑*, $\sigma_{t_{\text{swing}}}$↑, *σ*_DST_↑*	-1
U2: Walkers, ranging from non-wheeled to four-wheeled	Indicative evidence for reducing gait stability in non-impaired subjects	[85]	Crosswalker. Observational: walking speed reduced significantly.	9	λ of hip angle↑, λ of knee angle↑, λ of ankle angle↓, WS↓*, *l*_stride_↓*, Cadence↓*, *t*_stride_↑*, partial weight bearing↓	-1
		[85]	Four-wheeled walker. Convergent: Local dynamic stability reduced significantly.	9	λ of hip angle↑*, λ of knee angle↑*, λ of ankle angle↑*, WS↓*, *l*_stride_↓*, Cadence↓, *t*_stride_↑, partial weight bearing↓*	-1
		[86]	Four-wheeled walker. No significant findings reported	9	λ of 3D trunk acceleration~, *t*_stride_^*NR*^, WS^*NR*^, gait entropy, complexity, and determinism measures	0
		[86]	Motorized walker. No significant findings reported.	9	λ of 3D trunk acceleration~, *t*_stride_~, WS~, gait entropy, complexity, and determinism measures	0
U3: Handrail	Insufficient evidence for impaired subjects	[87]	A1. Observational: Stride time and step time symmetry increased significantly.	13	*t*_stride_↑*, $\sigma_{t_{\text{stride}}}$↓, *t*_step_△↑*, WS↑	+1
		[87]	A2. Observational: Stride time increased significantly.	13	*t*_stride_↑*, $\sigma_{t_{\text{stride}}}$↓, *t*_step_△↓, WS↓	0
U4: Forward force provided to the hand by a leash	Insufficient evidence for non-impaired subjects	[64]	B1. Observational: Walking speed and bilateral Sobolev norm difference did not change.	10	WS↑*, Sobolev norm~	+1
U4: Forward force provided to the hand by a leash	Insufficient evidence for impaired subjects	[64]	A1. Observational: Bilateral Sobolev norm difference increased significantly.	10	WS↑, Sobolev norm↑*	0
		[64]	A2. Observational: Bilateral Sobolev norm difference reduced significantly.	10	WS↑, Sobolev norm↓*	0

### Outcome measures

Across the 53 articles, we found more than 100 unique outcome measures, which were categorised into eleven categories based on their functional definitions, see Table 1. Due to this large number of measures, we denoted the outcome measure in Table 4 as denoted by the authors without providing separate definitions.

The six most commonly reported outcome measures were step width (SW) (n = 22), step length (SL) (n = 14), step width variability step width variability (*σ*_SW_) (n = 14), walking speed (n = 13), ML margin of stability (n = 11), and step length variability (*σ*_SL_) (n = 7). Of these only *σ*_SW_ and *σ*_SL_ are reported to have convergent validity. Lyapunov exponent (λ) was reported in ten papers but was calculated over 15 unique parameters (e.g. knee angle, trunk velocity, CoM pos). Whole-body centroidal angular momentum (*H*_r_) was reported only once (n = 1).

Due to the heterogeneity of the data and reported outcome measures, a meta-analysis was not possible.

Only within each large body-part category there was some consistency of reported outcome measures. Some examples: within the *Pelvis* group, *σ*_SW_ was measured 9 times, i.e. in 47% of the *Pelvis* studies. In all groups combined *σ*_SW_ was measured 14 times (26%). Similarly, WS was reported 9 times (82%) in the *Upper Extremity* group, compared to 13 times (25%) in all groups combined.

### Best-evidence synthesis

Fig 2 shows the overview of the level of evidence that was found for each intervention type, by visualising the various interventions, as depicted by their main force directions and device schematics. The intervention type codes (T1, P3, U2, etc.) are related to the matching codes in Table 4.

#### Trunk

From the included papers, eight involved trunk manipulation, describing five interventions. In non-impaired subjects, the most frequently used intervention was conventional body weight support (T1), which showed limited evidence for the improvement of gait stability [59, 61, 69–71]. Indicative evidence was provided in a single study applying both body weight support (BWS) and medio-lateral (ML) damping (T2) [61]. Two studies provided indicative evidence for improving gait stability by applying torques to the trunk (T3) [57, 72]. For impaired subjects, insufficient evidence was found on all intervention types.

#### Pelvis

19 papers involved force application on pelvis manipulation, describing five device types. The most frequently investigated device type contains ML forces applied to the pelvis (P1), for which moderate and indicative evidence was found, respectively, for non-impaired [16, 56, 58, 60, 65, 73–80] and impaired [60, 62, 73, 75, 78, 80–82] subjects. Other studies described BWS forces to the pelvis (P2), pelvis restriction devices (P3 and P4), and the effect of handrail combined with pelvis stabilisation (P5), providing insufficient evidence.

#### Upper extremity

Twelve papers involved force application on the upper extremity (hands/arms), describing four device types. Devices included the use of canes and walking sticks, walkers, handrails, and a leash. For walkers (U2), moderate and indicative evidence for reducing gait stability was found, respectively, for impaired subjects [83, 84] and non-impaired subjects [85, 86]. We found limited evidence for the improvement of gait stability with the use of walking-sticks and poles in impaired subjects (U1) [64, 84, 87–90]. In contrast, for non-impaired subjects indicative evidence for improving gait stability was found [64, 91, 92]. Other studies provided insufficient evidence.

#### Lower extremity

From the included articles, 16 involved force and/or torque application on the lower extremity, describing 6 device types, including external forces applied to the shank and powered hip-, knee- and ankle orthoses. Indicative evidence on improving gait stability was found for applying a knee-joint torque (L1) to non-impaired subjects [93–95]. Hip flexion assistance (L2) produced indicative and limited evidence respectively for non-impaired [96] and impaired subjects [54, 63, 66, 68]. The use of a powered ankle foot orthosis (AFO) (L6) shows contradictory findings for both impaired [55, 97] and non-impaired subjects [67, 98]. Other intervention types provided insufficient evidence.

## Discussion

### Main findings

For the impaired subjects, the highest level of evidence was found for ML pelvis stabilisation, showing indicative levels of evidence for improving gait stability. Limited evidence was found for hip joint stabilisation and canes. Interestingly, walkers produced a moderate level of evidence for *reducing* gait stability in impaired subjects. In non-impaired subjects, a moderate level of evidence was found for ML pelvis stabilisation, and limited evidence was found for body weight supports. For all other device types, at most, indicative evidence was found. Noteworthy is the indicative level of evidence that was found for *reducing* gait stability for hip joint assistance and walkers in non-impaired subjects. Due to the heterogeneity of the reported outcome measures, especially between groups, no meta-analysis was possible.

### Best-evidence synthesis

Finding a moderate level of evidence for (P1) ML pelvis manipulation of non-impaired subjects was not surprising, as this is generally assumed [16]. One reason might be that due to the proximity of the pelvis to the CoM, any forces applied to the pelvis almost directly influence CoM motion, the derivatives of which are major predictors of gait stability [3]. However, in impaired individuals, we unexpectedly found a lower level of evidence, mainly due to conflicting findings in the studies. Possibly disturbing a compensatory walking strategy in impaired subjects initially decreases gait stability, as adaptation periods are required before subjects utilise supporting forces and moments [106, 107]. All other articles related to pelvic manipulations were exploratory studies providing insufficient evidence. No studies were found that provided controlled AP or rotational support to the pelvis. This is possibly an interesting direction of study as manual rotational facilitation to the pelvis is used in the clinic to manipulate the gait of patients [108].

Concerning trunk manipulation, only the direct vertical body weight unloading method (T1) provided limited evidence for improving gait stability in non-impaired subjects. These findings are similar to findings reported by Apte et al. (2020) [109], although these effects might also be due to the medio-lateral centering effect of BWS systems [24]. BWS in combination with mediolateral damping to the pelvis (T2) provided indicative evidence. More indicative evidence was found for (T3) devices that apply moments to the trunk, such as backpacks containing gyroscopes or oscillating masses, though only few studies were found in this group. The strong link between the angular momentum of the body and gait stability makes this a promising direction of investigation [13].

A variety of interventions were used to manipulate the lower extremity. Due to differences between devices, each group contained few papers, generating limited evidence at best. Limited evidence was found for hip joint torque applied to the pelvis and thigh (L2) in impaired subjects. This is mainly caused by the limited use of outcome measures with convergent validity. Indicative evidence was found for devices that apply forces to the shank to manipulate the foot placement (L3). Foot placement is one of the critical elements of balance during walking [110]. The complexity of grabbing and manipulating the shank while in mid-air might explain the limited number of studies.

The studies that focused on the upper extremity generated contradictory results. For impaired subjects, the investigations on walking sticks provided only limited evidence for improving gait stability, mainly due to contradictory findings. A systematic review by Oates et al. (2017) described a reduction in variability of gait parameters and body stability as a result of haptic input of canes and handrails [111]. It is noteworthy that most of these papers did not measure or quantify the interaction forces between the subject and the device, making it difficult to replicate or compare their results. One clear finding is the evidence for the reduction of gait stability caused by walkers, in both the non-impaired and impaired groups. Walkers are known to alter posture and arm swing [84, 85, 112], thereby influencing overall gait stability.

Performing a study with non-impaired subjects is a logical first step in evaluating novel medical technology, as it is easier to obtain ethical approvals. This most likely explains their high occurrence in our review. Nevertheless, an investigation of improvements in gait stability in non-impaired people offers only very limited insights into the potential effects for rehabilitation. For example, our indicative finding that devices such as walking poles and sticks improve gait stability in non-impaired subjects does not provide a meaningful basis for any conclusions on the possible benefits of such devices for individuals who need assistance.

### Outcome measures

All studies combined reported over 100 unique outcome measures. For only three types of outcome measures, convergent validity is reported [3, 13, 15, 32, 45]. More than 40% percent of the papers rated eleven or higher, putting them in the upper ranges of our BES definition. This indicates that the low evidence levels found during our BES are mainly due to the lack of reported outcome measures with convergent validity and the large diversity of device types and not due to the quality of the studies.

The Lyapunov exponent (λ) is a widely accepted method of assessing gait stability [45]. The short-range λ has reported construct and predictive validity in models and convergent validity in experiments [3, 15]. In our review, it was reported in ten papers but was calculated over 15 unique parameters. This disparity in implementation and calculation was similarly concluded in a dedicated review by Mehdizadeh et al. (2018) [15]. We support their call for a standardisation of λ measurement and calculation across the field, for example, the Lyapunov exponent (λ) of the ML CoM position.

Kinematic variability is frequently used to assess gait stability [3, 14]. A reduction in variability, e.g. *σ*_SW_, is correlated with an improvement in gait stability [13, 110]. Nevertheless, evidence against a correlation between *σ*_SW_ and dynamic stability is also reported [59]. Step width variability and step length variability are among the most reported outcome measures in our study. Interestingly, these are hardly reported in studies assessing devices for the upper and lower extremities. One hypothesis is that studies regarding more traditional gait aid devices (i.e. canes, walkers, AFOs) are more focused on clinical outcome measures. This hypothesis is indirectly supported by the fact that the clinical measure walking speed is, conversely, almost never reported in the *Trunk* and *Pelvis* groups. As *σ*_SW_ is widely accepted and relatively easy to measure and calculate, we implore colleagues in the field to always report *σ*_SW_, or publish related raw data.

Even though MoS is an old [7] and widely used measure—it is among the most reported in our study—convergent validity in experimental studies seems to be lacking [3, 32, 37, 38] and differences in methodology and interpretation still seem to hinder comparison between studies [20].

### Preregistration of studies

In the pool of included studies, the number of papers with a preregistered protocol and outcome measures is very low (< 6%). The absence of preregistration potentially allows researchers to change reported outcome measures after the data is retrieved, increasing the risk of p-hacking and cherry-picking [113, 114]. With websites such as https://osf.io/ [115] the process is fairly straightforward. Thus, we advise researchers to take this step into account before performing their study.

### Limitations

Gait stability is a wide term encompassing the human ability to recover from 1) minor cyclic perturbations that occur every step, 2) large perturbations that require a change in overall walking pattern, and 3) the largest recoverable perturbation [3]. In our study, we focused mainly on minor perturbations.

During our analyses, we grouped devices based on the main point of application of their forces and their directions. Such a simplification is insufficient to fully describe how a device works regarding weight shifting, postural restrictions, and even a perceived sense of safety. Regardless, some grouping was required to provide a broad view of the sensitivity of gait stability to forces applied across varying points of applications. Additionally, when running simulations of gait manipulations, for instance in SCONE [116], the effect of a particular added force or moment is similarly distilled into a single point or segment of application, respectively.

Not all studies primarily aimed at gait stability manipulation, or used the term ‘gait stability’, which likely influenced their choice in reported outcome measures and influenced their comparability. Similarly, grouping all impaired subjects for the comparisons within each device type limits the strength of the conclusions that can be drawn. However, the presented cross-disorder conclusions on the direction of devices can still be justified, as we specifically looked at studies containing a baseline and direct intervention measurement.

### Outlook

Our main findings can possibly be translated into further improvement of rehabilitation devices and aids. For instance, the evidence surrounding lateral stabilisation of the pelvis could be used in concert with the pelvis-manipulating active device MUCDA [117] or with cold-gas thrusters [25] to provide lateral damping forces. Similarly, the finding that walkers seem to cause a reduction in gait stability could warrant further investigation into the long-term effects of walker use.

Comparison among devices is hindered by the absence of a gold standard and the heterogeneity of the reported outcome measures. We advise using at least (one of) the following outcome measures: short-range Lyapunov exponent (λ), step width variability (*σ*_SW_), and whole-body centroidal angular momentum (*H*_r_). Furthermore, we implore researchers to preregister their trials to reduce the risk of cherry-picking and to share original data that would allow them to recalculate the above-mentioned outcome measures.

## Conclusion

The best evidence synthesis found at most moderate evidence for any intervention. A moderate level of evidence was found for direct improvement of gait stability due to mediolateral pelvis manipulation for non-impaired subjects. Torques applied to the hip joint, and walking poles, sticks, canes and crutches only showed limited evidence for improving gait stability for impaired subjects. Promising, indicative evidence was found for torques applied to the trunk. Moderate and indicative evidence was found for *reducing* gait stability for walkers for impaired and non-impaired subjects, respectively. Our findings also highlight the lack of consensus on outcome measures amongst studies of devices focused on manipulating gait.

## Supporting information

S1 Appendix Search strategy. The full search strategy for each database can be found in this supporting document.
(PDF)

S1 Table Quality and risk of bias assessment tool. The modified NIH quality and risk of bias assessment tool can be found here.
(PDF)

S2 Table Quality and risk of bias scores. The full results of the quality and risk of bias assessment tool, for each article, can be found here.
(PDF)

S1 Checklist PRISMA 2009 checklist. (PDF)

## Abbreviations

λ: Lyapunov exponent
△: symmetry
6MWT: six minute walking test
$\sigma_{l_{\text{stride}}}$: stride length variability
$\sigma_{t_{\text{stride}}}$: stride time variability
$\sigma_{t_{\text{swing}}}$: swing time variability
*θ*: angle
*σ*: variability
*σ*_DST_: double support time variability
*σ*_SL_: step length variability
*σ*_ST_: step time variability
*σ*_SW_: step width variability
*d*_CoM,ML_: CoM medio-lateral displacement
*H*_r_: whole-body centroidal angular momentum
*l*_stride_: stride length
*r*_CoM,ML_: CoM medio-lateral position
*r*_FP,ML_: medio-lateral foot placement
*t*_stance_: stance time
*t*_step_: step time
*t*_stride_: stride time
*t*_swing_: swing time
*v*_CoM,ML_: CoM medio-lateral velocity
AP: anterior-posterior
BES: best-evidence synthesis
BoS: base of support
BWS: body weight support
CoM: center of mass
DST: double support time
FM: Floquet multiplier
GCT: gait cycle time
HS: heel strike
MAD: median absolute deviation
ML: medio-lateral
MoS: margin of stability
NIH: National Institutes of Health
RoM: range of motion
SH: step height
SL: step length
SST: single support time
SW: step width
WS: walking speed
XCoM: extrapolated center of mass

## References

[1] Rubenstein LZ. Falls in older people: epidemiology, risk factors and strategies for prevention. Age and Ageing. 2006;35(suppl_2):ii37–ii41. doi: 10.1093/ageing/afl084 16926202

[2] Berg WP, Alessio HM, Mills EM, Tong C. Correlates of Recurrent Falling in Independent Community-Dwelling Older Adults. Journal of Motor Behavior. 1997;29(1):5–16. doi: 10.1080/00222899709603465 20037005

[3] Bruijn SM, Meijer OG, Beek PJ, van Dieën JH. Assessing the stability of human locomotion: a review of current measures. Journal of The Royal Society Interface. 2013;10(83):20120999. doi: 10.1098/rsif.2012.0999 23516062 PMC3645408

[4] Osoba MY, Rao AK, Agrawal SK, Lalwani AK. Balance and gait in the elderly: A contemporary review: Balance and Gait in the Elderly. Laryngoscope Investigative Otolaryngology. 2019;4(1):143–153. doi: 10.1002/lio2.252 30828632 PMC6383322

[5] Schniepp R, Huppert A, Decker J, Schenkel F, Schlick C, Rasoul A, et al. Fall prediction in neurological gait disorders: differential contributions from clinical assessment, gait analysis, and daily-life mobility monitoring;268(9):3421–3434. doi: 10.1007/s00415-021-10504-x 33713194 PMC8357767

[6] Pratt JE, Tedrake R. Velocity-Based Stability Margins for Fast Bipedal Walking. In: Diehl M, Mombaur K, editors. Fast Motions in Biomechanics and Robotics. vol. 340. Berlin, Heidelberg: Springer Berlin Heidelberg; 2006. p. 299–324. Available from: http://link.springer.com/10.1007/978-3-540-36119-0_14.

[7] Hof AL, Gazendam MGJ, Sinke WE. The condition for dynamic stability. Journal of Biomechanics. 2005;38(1):1–8. doi: 10.1016/j.jbiomech.2004.03.025 15519333

[8] Shirota C, van Asseldonk E, Matjačić Z, Vallery H, Barralon P, Maggioni S, et al. Robot-supported assessment of balance in standing and walking. Journal of NeuroEngineering and Rehabilitation. 2017;14(1):80. doi: 10.1186/s12984-017-0273-7 28806995 PMC5556664

[9] England SA, Granata KP. The influence of gait speed on local dynamic stability of walking. Gait & posture. 2007;25(2):172–178. doi: 10.1016/j.gaitpost.2006.03.003 16621565 PMC1785331

[10] Roeles S, Rowe PJ, Bruijn SM, Childs CR, Tarfali GD, Steenbrink F, et al. Gait stability in response to platform, belt, and sensory perturbations in young and older adults. Medical & Biological Engineering & Computing. 2018;56(12):2325–2335. doi: 10.1007/s11517-018-1855-7 29946955 PMC6245003

[11] Mombaur K, Vallery H, Hu Y, Buchli J, Bhounsule P, Boaventura T, et al. Control of Motion and Compliance. Bioinspired Legged Locomotion. 2017; p. 135–346. doi: 10.1016/B978-0-12-803766-9.00006-3

[12] Kuo AD, Donelan JM. Dynamic Principles of Gait and Their Clinical Implications. Physical Therapy. 2010;90(2):157–174. doi: 10.2522/ptj.20090125 20023002 PMC2816028

[13] Neptune RR, Vistamehr A. Dynamic Balance During Human Movement: Measurement and Control Mechanisms. Journal of Biomechanical Engineering. 2019;141(7):070801. doi: 10.1115/1.4042170 PMC6611347 30516241

[14] Hamacher D, Singh NB, Van Dieën JH, Heller MO, Taylor WR. Kinematic measures for assessing gait stability in elderly individuals: a systematic review. Journal of The Royal Society Interface. 2011;8(65):1682–1698. doi: 10.1098/rsif.2011.0416 21880615 PMC3203491

[15] Mehdizadeh S. The largest Lyapunov exponent of gait in young and elderly individuals: A systematic review. Gait & Posture. 2018;60:241–250. doi: 10.1016/j.gaitpost.2017.12.016 29304432

[16] Mahaki M, IJ T, Houdijk H, Bruijn SM. How does external lateral stabilization constrain normal gait, apart from improving medio-lateral gait stability? R Soc open sci. 2021;8(3):202088. doi: 10.1098/rsos.202088 33959361 PMC8074891

[17] Alizadehsaravi L, Bruijn SM, Muijres W, Koster RAJ, Van Dieën JH. Improvement in gait stability in older adults after ten sessions of standing balance training;17(7):e0242115. doi: 10.1371/journal.pone.0242115 35895709 PMC9328559

[18] Matjačić Z, Zadravec M, Olenšek A. Feasibility of robot-based perturbed-balance training during treadmill walking in a high-functioning chronic stroke subject: a case-control study. Journal of NeuroEngineering and Rehabilitation. 2018;15(1):32. doi: 10.1186/s12984-018-0373-z 29642921 PMC5896154

[19] McCrum C, Gerards MHG, Karamanidis K, Zijlstra W, Meijer K. A systematic review of gait perturbation paradigms for improving reactive stepping responses and falls risk among healthy older adults. European Review of Aging and Physical Activity. 2017;14(1):3. doi: 10.1186/s11556-017-0173-7 28270866 PMC5335723

[20] Watson F, Fino PC, Thornton M, Heracleous C, Loureiro R, Leong JJH. Use of the margin of stability to quantify stability in pathologic gait—a qualitative systematic review. BMC Musculoskeletal Disorders. 2021;22(1):597. doi: 10.1186/s12891-021-04466-4 34182955 PMC8240253

[21] Hamacher D, Liebl D, Hödl C, Heßler V, Kniewasser CK, Thönnessen T, et al. Gait Stability and Its Influencing Factors in Older Adults. Frontiers in Physiology. 2019;9:1955. doi: 10.3389/fphys.2018.01955 30733686 PMC6354563

[22] Lee Y, Curuk E, Aruin AS. Effect of Light Finger Touch, a Cognitive Task, and Vision on Standing Balance in Stroke;53(2):157–165. doi: 10.1080/00222895.2020.1742082 32281912

[23] Sienko KH, Seidler RD, Carender WJ, Goodworth AD, Whitney SL, Peterka RJ. Potential Mechanisms of Sensory Augmentation Systems on Human Balance Control;9:944. doi: 10.3389/fneur.2018.00944 30483209 PMC6240674

[24] Plooij M, Apte S, Keller U, Baines P, Sterke B, Asboth L, et al. Neglected physical human-robot interaction may explain variable outcomes in gait neurorehabilitation research. Science Robotics. 2021;6(58):eabf1888. doi: 10.1126/scirobotics.abf1888 34550719

[25] Baimyshev A, Finn-Henry M, Goldfarb M. Feasibility of a Wearable Cold-Gas Thruster for Fall Prevention;144(8):084501. doi: 10.1115/1.4054529

[26] Peters DM, O’Brien ES, Kamrud KE, Roberts SM, Rooney TA, Thibodeau KP, et al. Utilization of wearable technology to assess gait and mobility post-stroke: a systematic review. Journal of NeuroEngineering and Rehabilitation. 2021;18(1):67. doi: 10.1186/s12984-021-00863-x 33882948 PMC8059183

[27] Slavin RE. Best-Evidence Synthesis: An Alternative to Meta-Analytic and Traditional Reviews. Educational Researcher. 1986. doi: 10.3102/0013189X015009005

[28] Bramer WM, Rethlefsen ML, Mast F, Kleijnen J. Evaluation of a new method for librarian-mediated literature searches for systematic reviews. Research Synthesis Methods. 2018;9(4):510–520. doi: 10.1002/jrsm.1279 29073718 PMC5920798

[29] Page MJ, McKenzie JE, Bossuyt PM, Boutron I, Hoffmann TC, Mulrow CD, et al. The PRISMA 2020 statement: an updated guideline for reporting systematic reviews. Systematic Reviews. 2021;10(1):89. doi: 10.1186/s13643-021-01626-4 33781348 PMC8008539

[30] Middleton A, Fritz SL. Assessment of Gait, Balance, and Mobility in Older Adults: Considerations for Clinicians. Current Translational Geriatrics and Experimental Gerontology Reports. 2013;2(4):205–214. doi: 10.1007/s13670-013-0057-2

[31] Stokes HE, Thompson JD, Franz JR. The Neuromuscular Origins of Kinematic Variability during Perturbed Walking. Scientific Reports. 2017;7(1):808. doi: 10.1038/s41598-017-00942-x 28400615 PMC5429788

[32] Vistamehr A, Kautz SA, Bowden MG, Neptune RR. Correlations between measures of dynamic balance in individuals with post-stroke hemiparesis. Journal of Biomechanics. 2016;49(3):396–400. doi: 10.1016/j.jbiomech.2015.12.047 26795124 PMC4761510

[33] Nott CR, Neptune RR, Kautz SA. Relationships between frontal-plane angular momentum and clinical balance measures during post-stroke hemiparetic walking. Gait & Posture. 2014;39(1):129–134. doi: 10.1016/j.gaitpost.2013.06.008 23820449 PMC3823741

[34] Bhat SG, Subramanian SC, Sugar TS, Redkar S. Application of Floquet Theory to Human Gait Kinematics and Dynamics. Journal of Mechanisms and Robotics. 2021;13(6):061003. doi: 10.1115/1.4050199

[35] Wodarski P, Jurkojć J, Polechoński J, Bieniek A, Chrzan M, Michnik R, et al. Assessment of gait stability and preferred walking speed in virtual reality. Acta of Bioengineering and Biomechanics. 2020;22(1). doi: 10.37190/ABB-01490-2019-03 32307457

[36] Riva F, Toebes MJP, Pijnappels M, Stagni R, van Dieën JH. Estimating fall risk with inertial sensors using gait stability measures that do not require step detection. Gait & Posture. 2013;38(2):170–174. doi: 10.1016/j.gaitpost.2013.05.002 23726429

[37] Hurt CP, Grabiner MD. Age-related differences in the maintenance of frontal plane dynamic stability while stepping to targets. Journal of Biomechanics. 2015;48(4):592–597. doi: 10.1016/j.jbiomech.2015.01.003 25627870 PMC4362666

[38] Kao PC, Dingwell JB, Higginson JS, Binder-Macleod S. Dynamic instability during post-stroke hemiparetic walking. Gait & Posture. 2014;40(3):457–463. doi: 10.1016/j.gaitpost.2014.05.014 24931112 PMC4251664

[39] Kim JH. Relationship between gait symmetry and functional balance, walking performance in subjects with stroke. The Journal of Korean Physical Therapy. 2014;26(1):1–8. doi: 10.18857/jkpt.2015.27.1.1

[40] An CM, Son YL, Park YH, Moon SJ. Relationship between dynamic balance and spatiotemporal gait symmetry in hemiplegic patients with chronic stroke. Hong Kong Physiotherapy Journal. 2017;37:19–24. doi: 10.1016/j.hkpj.2017.01.002 30931042 PMC6385150

[41] Lockhart TE, Liu J. Differentiating fall-prone and healthy adults using local dynamic stability. Ergonomics. 2008;51(12):1860–1872. doi: 10.1080/00140130802567079 19034782 PMC2892176

[42] Herman T, Giladi N, Gurevich T, Hausdorff JM. Gait instability and fractal dynamics of older adults with a “cautious” gait: why do certain older adults walk fearfully? Gait & Posture. 2005;21(2):178–185. doi: 10.1016/j.gaitpost.2004.01.014 15639397

[43] Pijnappels M, van der Burg PJCE, Reeves ND, van Dieën JH. Identification of elderly fallers by muscle strength measures. European Journal of Applied Physiology. 2008;102(5):585–592. doi: 10.1007/s00421-007-0613-6 18071745 PMC2226001

[44] Dingwell JB. Lyapunov Exponents. In: Wiley Encyclopedia of Biomedical Engineering. John Wiley & Sons, Ltd; 2006. Available from: https://onlinelibrary.wiley.com/doi/abs/10.1002/9780471740360.ebs0702.

[45] Dingwell JB, Cusumano JP, Cavanagh PR, Sternad D. Local Dynamic Stability Versus Kinematic Variability of Continuous Overground and Treadmill Walking. Journal of Biomechanical Engineering. 2001;123(1):27–32. doi: 10.1115/1.1336798 11277298

[46] Herr H, Popovic M. Angular momentum in human walking. Journal of Experimental Biology. 2008;211(4):467–481. doi: 10.1242/jeb.008573 18245623

[47] Neptune RR, McGowan CP. Muscle contributions to frontal plane angular momentum during walking. Journal of Biomechanics. 2016;49(13):2975–2981. doi: 10.1016/j.jbiomech.2016.07.016 27522538 PMC5056157

[48] Neptune RR, McGowan CP. Muscle contributions to whole-body sagittal plane angular momentum during walking. Journal of Biomechanics. 2011;44(1):6–12. doi: 10.1016/j.jbiomech.2010.08.015 20833396 PMC3003775

[49] Hof AL. The ‘extrapolated center of mass’ concept suggests a simple control of balance in walking. Human Movement Science. 2008;27(1):112–125. doi: 10.1016/j.humov.2007.08.003 17935808

[50] Cromwell RL, Newton RA. Relationship between balance and gait stability in healthy older adults. Journal of Aging & Physical Activity. 2004;12(1). doi: 10.1123/japa.12.1.90 15211023

[51] Balasubramanian CK, Neptune RR, Kautz SA. Variability in spatiotemporal step characteristics and its relationship to walking performance post-stroke. Gait & Posture. 2009;29(3):408–414. doi: 10.1016/j.gaitpost.2008.10.061 19056272 PMC2675553

[52] Verlinden VJA, van der Geest JN, Hoogendam YY, Hofman A, Breteler MMB, Ikram MA. Gait patterns in a community-dwelling population aged 50 years and older. Gait & Posture. 2013;37(4):500–505. doi: 10.1016/j.gaitpost.2012.09.005 23018028

[53] National Heart aBIN Lung. Quality Assessment Tool for Before-After (Pre-Post) Studies With No Control Group; 2021. Available from: https://www.nhlbi.nih.gov/health-topics/study-quality-assessment-tools.

[54] Lee SH, Lee HJ, Chang WH, Choi BO, Lee J, Kim J, et al. Gait performance and foot pressure distribution during wearable robot-assisted gait in elderly adults. J NeuroEng Rehabil. 2017;14(1). doi: 10.1186/s12984-017-0333-z 29183379 PMC5706419

[55] Hsu CC, Huang YK, Kang JH, Ko YF, Liu CW, Jaw FS, et al. Novel design for a dynamic ankle foot orthosis with motion feedback used for training in patients with hemiplegic gait: A pilot study. J NeuroEng Rehabil. 2020;17(1). doi: 10.1186/s12984-020-00734-x 32811516 PMC7433152

[56] Mahaki M, Bruijn SM, Van Dieën JH. The effect of external lateral stabilization on the use of foot placement to control mediolateral stability in walking and running. PeerJ. 2019;2019(10). doi: 10.7717/peerj.7939 31681515 PMC6822599

[57] Lemus D, Berry A, Jabeen S, Jayaraman C, Hohl K, van der Helm FCT, et al. Controller synthesis and clinical exploration of wearable gyroscopic actuators to support human balance. Sci rep. 2020;10(1):10412. doi: 10.1038/s41598-020-66760-w 32591577 PMC7320159

[58] Bruijn SM, Van Dieën JH, Daffertshofer A. Beta activity in the premotor cortex is increased during stabilized as compared to normal walking. Front Human Neurosci. 2015;9(OCTOBER). doi: 10.3389/fnhum.2015.00593 26578937 PMC4621867

[59] Ignasiak NK, Ravi DK, Orter S, Nasab SHH, Taylor WR, Singh NB. Does variability of footfall kinematics correlate with dynamic stability of the centre of mass during walking? PLoS ONE. 2019;14(5). doi: 10.1371/journal.pone.0217460 PMC6544240 31150452

[60] Ochs WL, Woodward J, Cornwell T, Gordon KE. Meaningful measurements of maneuvers: People with incomplete spinal cord injury’step up’ to the challenges of altered stability requirements. J Neuroengineering Rehabil. 2021;18(1):46. doi: 10.1186/s12984-021-00840-4 PMC7927389 33653370

[61] Bannwart M, Bayer SL, König Ignasiak N, Bolliger M, Rauter G, Easthope CA. Mediolateral damping of an overhead body weight support system assists stability during treadmill walking. J NeuroEng Rehabil. 2020;17(1). doi: 10.1186/s12984-020-00735-w 32778127 PMC7418206

[62] Lin JT, Hsu CJ, Dee W, Chen D, Rymer WZ, Wu M. Error variability affects the after effects following motor learning of lateral balance control during walking in people with spinal cord injury. Eur J Neurosci. 2019;50(8):3221–3234. doi: 10.1111/ejn.14478 31161634 PMC6821555

[63] Monaco V, Tropea P, Aprigliano F, Martelli D, Parri A, Cortese M, et al. An ecologically-controlled exoskeleton can improve balance recovery after slippage. Sci rep. 2017;7:46721. doi: 10.1038/srep46721 28492520 PMC5426188

[64] Sorrento GU, Archambault PS, Fung J. Walking with robot-generated haptic forces in a virtual environment: a new approach to analyze lower limb coordination. J NeuroEng Rehabil. 2021;18(1). doi: 10.1186/s12984-021-00823-5 34503526 PMC8428107

[65] Walker ER, Hyngstrom AS, Onushko T, Schmit BD. Locomotor adaptations to prolonged step-by-step frontal plane trunk perturbations in young adults. PLoS ONE. 2018;13(9). doi: 10.1371/journal.pone.0203776 30235250 PMC6147485

[66] Livolsi C, Conti R, Guanziroli E, Fririksson, Alexandersson A, Kristjansson K, et al. An impairment-specific hip exoskeleton assistance for gait training in subjects with acquired brain injury: a feasibility study. Sci rep. 2022;12(1):19343. doi: 10.1038/s41598-022-23283-w 36369462 PMC9652374

[67] Choi HS, Baek YS, In H. Ankle strategy assistance to improve gait stability using controllers based on in-shoe center of pressure in 2 degree-of-freedom powered ankle-foot orthoses: a clinical study. J Neuroengineering Rehabil. 2021;19(1):114. doi: 10.1186/s12984-022-01092-6 PMC9594937 36284358

[68] Qian Y, Yu H, Fu C. Adaptive Oscillator-Based Assistive Torque Control for Gait Asymmetry Correction With a nSEA-Driven Hip Exoskeleton. IEEE Trans Neural Syst Rehabil Eng. 2022;30:2906–2915. doi: 10.1109/TNSRE.2022.3213810 36223362

[69] Goncalves RS, Krebs HI. MIT-Skywalker: considerations on the design of a body weight support system. J Neuroengineering Rehabil. 2017;14(1):88. doi: 10.1186/s12984-017-0302-6 28877750 PMC5588735

[70] Dragunas AC, Gordon KE. Body weight support impacts lateral stability during treadmill walking. Journal of Biomechanics. 2016;49(13):2662–2668. doi: 10.1016/j.jbiomech.2016.05.026 27282960 PMC5056129

[71] Pillar T, Dickstein R, Smolinski Z. Walking reeducation with partial relief of body weight in rehabilitation of patients with locomotor disabilities. J REHABIL RES DEV. 1991;28(4):47–52. doi: 10.1682/JRRD.1991.10.0047 1941649

[72] Best AN, Martin JP, Li Q, Wu AR. Stepping behaviour contributes little to balance control against continuous mediolateral trunk perturbations. J Exp Biol. 2019;222. doi: 10.1242/jeb.212787 31767732

[73] Dean JC, Alexander NB, Kuo AD. The effect of lateral stabilization on walking in young and old adults. IEEE Transactions on Biomedical Engineering. 2007;54(11):1919–1926. doi: 10.1109/TBME.2007.901031 18018687

[74] Donelan JM, Shipman DW, Kram R, Kuo AD. Mechanical and metabolic requirements for active lateral stabilization in human walking. Journal of Biomechanics. 2004;37(6):827–835. doi: 10.1016/j.jbiomech.2003.06.002 15111070

[75] Frame HB, Finetto C, Dean JC, Neptune RR. The influence of lateral stabilization on walking performance and balance control in neurologically-intact and post-stroke individuals. Clinical Biomechanics. 2020;73:172–180. doi: 10.1016/j.clinbiomech.2020.01.005 32004909 PMC7183884

[76] Ijmker T, Houdijk H, Lamoth CJC, Beek PJ, van der Woude LHV. Energy cost of balance control during walking decreases with external stabilizer stiffness independent of walking speed. Journal of Biomechanics. 2013;46(13):2109–2114. doi: 10.1016/j.jbiomech.2013.07.005 23895896

[77] Koopman B, Meuleman JH, van Asseldonk EH, van der Kooij H. Lateral balance control for robotic gait training. IEEE Int Conf Rehabil Robot. 2013;2013:6650363. doi: 10.1109/icorr.2013.6650363 24187182

[78] Wu M, Brown G, Gordon KE. Control of locomotor stability in stabilizing and destabilizing environments. Gait and Posture. 2017;55:191–198. doi: 10.1016/j.gaitpost.2017.04.021 28477529

[79] van Leeuwen AM, van Dieen JH, Bruijn SM. The effect of external lateral stabilization on ankle moment control during steady-state walking. J Biomech. 2022;142:111259. doi: 10.1016/j.jbiomech.2022.111259 36027635

[80] Dragunas AC, Cornwell T, Lopez-Rosado R, Gordon KE. Post-Stroke Adaptation of Lateral Foot Placement Coordination in Variable Environments. IEEE Trans Neural Syst Rehabil Eng. 2021;29:731–739. doi: 10.1109/TNSRE.2021.3072252 33835919 PMC8115208

[81] Matsubara JH, Wu M, Gordon KE. Metabolic cost of lateral stabilization during walking in people with incomplete spinal cord injury. Gait and Posture. 2015;41(2):646–651. doi: 10.1016/j.gaitpost.2015.01.015 25670651 PMC4318628

[82] Lin JT, Hsu CJ, Dee W, Chen D, Rymer WZ, Wu M. Motor Adaptation to Weight Shifting Assistance Transfers to Overground Walking in People with Spinal Cord Injury. Pm R. 2019;11(11):1200–1209. doi: 10.1002/pmrj.12132 30729754 PMC6685757

[83] Zhang M, Artan NS, Gu H, Dong Z, Burina Ganatra L, Shermon S, et al. Gait study of parkinson’s disease subjects using haptic cues with a motorized walker. Sensors (Basel). 2018;18(10). doi: 10.3390/s18103549 30347753 PMC6210411

[84] Kloos AD, Kegelmeyer DA, White SE, Kostyk SK. The impact of different types of assistive devices on gait measures and safety in Huntington’s disease. PLoS ONE. 2012;7(2). doi: 10.1371/journal.pone.0030903 22363511 PMC3281896

[85] Seiferheld BE, Frost J, Andersen C, Samani A. New assistive walker improved local dynamic stability in young healthy adults. J Electromyogr Kinesiology. 2020;53. doi: 10.1016/j.jelekin.2020.102441 32629410

[86] Wan X, Yamada Y. An Acceleration-Based Nonlinear Time-Series Analysis of Effects of Robotic Walkers on Gait Dynamics During Assisted Walking. IEEE Sensors Journal. 2022;. doi: 10.1109/JSEN.2022.3206545

[87] Ijmker T, Houdijk H, Lamoth CJ, Jarbandhan AV, Rijntjes D, Beek PJ, et al. Effect of balance support on the energy cost of walking after stroke. Archives of Physical Medicine and Rehabilitation. 2013;94(11):2255–2261. doi: 10.1016/j.apmr.2013.04.022 23702394

[88] Sonntag D, Uhlenbrock D, Bardeleben A, Kading M, Hesse S. Gait with and without forearm crutches in patients with total hip arthroplasty. International Journal of Rehabilitation Research. 2000;23(3):233–243. doi: 10.1097/00004356-200023030-00014 11131626

[89] Polese JC, Teixeira-Salmela LF, Nascimento LR, Faria CDM, Kirkwood RN, Laurentino GC, et al. The effects of walking sticks on gait kinematics and kinetics with chronic stroke survivors. Clinical Biomechanics. 2012;27(2):131–137. doi: 10.1016/j.clinbiomech.2011.08.003 21889240

[90] Maguire C, Sieben JM, Frank M, Romkes J. Hip abductor control in walking following stroke—the immediate effect of canes, taping and TheraTogs on gait. Clin Rehabil. 2010;24(1):37–45. doi: 10.1177/0269215509342335 19906767

[91] Jayakaran P, DeSouza L, Cossar J, Gilhooly K. Influence of a walking aid on temporal and spatial parameters of gait in healthy adults. PM R. 2014;6(9):796–801. doi: 10.1016/j.pmrj.2014.02.005 24534098

[92] Zoffoli L, Ditroilo M, Federici A, Lucertini F. Local stability and kinematic variability in walking and pole walking at different speeds. Gait and Posture. 2017;53:1–4. doi: 10.1016/j.gaitpost.2016.12.017 28061400

[93] Chinimilli PT, Rezayat Sorkhabadi SM, Zhang W. Assessment of human dynamic gait stability with a lower extremity assistive device. IEEE transactions on neural systems and rehabilitation engineering: a publication of the IEEE Engineering in Medicine and Biology Society. 2020;28(3):669–678. doi: 10.1109/TNSRE.2020.2970207 32011260

[94] Haufe FL, Kober AM, Wolf P, Riener R, Xiloyannis M. Learning to walk with a wearable robot in 880 simple steps: a pilot study on motor adaptation. J NeuroEng Rehabil. 2021;18(1). doi: 10.1186/s12984-021-00946-9 34724940 PMC8561899

[95] Bacek T, Moltedo M, Serrien B, Langlois K, Vanderborght B, Lefeber D, et al. Human Musculoskeletal and Energetic Adaptations to Unilateral Robotic Knee Gait Assistance. IEEE Trans Biomed Eng. 2022;69(3):1141–1150. doi: 10.1109/TBME.2021.3114737 34559629

[96] Park JH, Kim S, Nussbaum MA, Srinivasan D. Effects of back-support exoskeleton use on gait performance and stability during level walking. Gait Posture. 2022;92:181–190. doi: 10.1016/j.gaitpost.2021.11.028 34864386

[97] Galle S, Derave W, Bossuyt F, Calders P, Malcolm P. Exoskeleton plantarflexion assistance for elderly. Gait & Posture. 2017;52:183–188. doi: 10.1016/j.gaitpost.2016.11.040 27915222

[98] Norris JA, Marsh AP. Positive feedback in powered exoskeletons: Improved metabolic efficiency at the cost of reduced stability? 6th International Conference on Multibody Systems, Nonlinear Dynamics, and Control. 2007;pp. 1619-1626. ASME.

[99] Clark RA, Williams G, Fini N, Moore L, Bryant AL. Coordination of dynamic balance during gait training in people with acquired brain injury. Archives of Physical Medicine and Rehabilitation. 2012;93(4):636–640. doi: 10.1016/j.apmr.2011.11.002 22325681

[100] Heitkamp LN, Stimpson KH, Dean JC. Application of a Novel Force-Field to Manipulate the Relationship between Pelvis Motion and Step Width in Human Walking. IEEE Trans Neural Syst Rehabil Eng. 2019;27(10):2051–2058. doi: 10.1109/TNSRE.2019.2941372 31545734

[101] Nyberg ET, Broadway J, Finetto C, Dean JC. A Novel Elastic Force-Field to Influence Mediolateral Foot Placement during Walking. IEEE Trans Neural Syst Rehabil Eng. 2017;25(9):1481–1488. doi: 10.1109/TNSRE.2016.2633960 27913354

[102] Reimold NK, Knapp HA, Chesnutt AN, Agne A, Dean JC. Effects of Targeted Assistance and Perturbations on the Relationship between Pelvis Motion and Step Width in People with Chronic Stroke. IEEE Trans Neural Syst Rehabil Eng. 2021;29:134–143. doi: 10.1109/TNSRE.2020.3038173 33196440 PMC8844911

[103] Yen SC, Schmit BD, Wu M. Using swing resistance and assistance to improve gait symmetry in individuals post-stroke. Human movement science. 2015;42:212–224. doi: 10.1016/j.humov.2015.05.010 26066783 PMC4508206

[104] Vashista V, Agrawal N, Shaharudin S, Reisman DS, Agrawal SK. Force adaptation in human walking with symmetrically applied downward forces on the pelvis. Annu Int Conf IEEE Eng Med Biol Soc. 2013;2013:3312–3315. doi: 10.1109/embc.2012.6346673 23366634

[105] Graham SA, Hurt CP, Brown DA. Minimizing postural demands of walking while still emphasizing locomotor force generation for nonimpaired individuals. IEEE transactions on neural systems and rehabilitation engineering: a publication of the IEEE Engineering in Medicine and Biology Society. 2018;26(5):1003–1010. doi: 10.1109/TNSRE.2018.2810701 29752235 PMC7968074

[106] Bastian AJ. Understanding sensorimotor adaptation and learning for rehabilitation. Current Opinion in Neurology. 2008;21(6):628–633. doi: 10.1097/WCO.0b013e328315a293 18989103 PMC2954436

[107] Poggensee KL, Collins SH. How adaptation, training, and customization contribute to benefits from exoskeleton assistance. SCIENCE ROBOTICS. 2021; p. 14. doi: 10.1126/scirobotics.abf1078 34586837

[108] Son C, Lee A, Lee J, Kim D, Kim SJ, Chun MH, et al. The effect of pelvic movements of a gait training system for stroke patients: a single blind, randomized, parallel study. Journal of NeuroEngineering and Rehabilitation. 2021;18(1):185. doi: 10.1186/s12984-021-00964-7 34961541 PMC8714451

[109] Apte S, Plooij M, Vallery H. Influence of body weight unloading on human gait characteristics: a systematic review. Journal of NeuroEngineering and Rehabilitation. 2018;15(1):53. doi: 10.1186/s12984-018-0380-0 29925400 PMC6011391

[110] McAndrew Young PM, Dingwell JB. Voluntarily changing step length or step width affects dynamic stability of human walking. Gait & Posture. 2012;35(3):472–477. doi: 10.1016/j.gaitpost.2011.11.010 22172233 PMC3299923

[111] Oates AR, Hauck L, Moraes R, Sibley KM. The effects of haptic input on biomechanical and neurophysiological parameters of walking: A scoping review. Gait & Posture. 2017;58:232–239. doi: 10.1016/j.gaitpost.2017.08.004 28822328

[112] Martins M, Santos C, Frizera A, Ceres R. A review of the functionalities of smart walkers. Medical Engineering & Physics. 2015;37(10):917–928. doi: 10.1016/j.medengphy.2015.07.006 26307456

[113] Mertens G, Krypotos AM. Preregistration of Analyses of Preexisting Data. Psychologica Belgica. 2019;59(1):338–352. doi: 10.5334/pb.493 31497308 PMC6706998

[114] Sijtsma K, Emons WHM, Steneck NH, Bouter LM. Steps toward preregistration of research on research integrity. Research Integrity and Peer Review. 2021;6(1):5. doi: 10.1186/s41073-021-00108-4 33648609 PMC7923522

[115] for Open Science C. The Open Science Framework; 2023. Available from: https://osf.io/.

[116] Geijtenbeek T. SCONE: Open Source Software for Predictive Simulation of Biological Motion;4(38):1421. doi: 10.21105/joss.01421

[117] Wyss D, Pennycott A, Bartenbach V, Riener R, Vallery H. A MUltidimensional Compliant Decoupled Actuator (MUCDA) for Pelvic Support During Gait. IEEE/ASME Transactions on Mechatronics. 2019;24(1):164–174. doi: 10.1109/TMECH.2018.2878289

