# Supplementary material for: Direct biomechanical manipulation of human gait stability: A systematic review
Source: PLoS One. 2024 Jul 11;19(7):e0305564. doi: 10.1371/journal.pone.0305564 (PMC11239080; doi:10.1371/journal.pone.0305564)
Supplement: S1 Appendix — The full search strategy for each database can be found in this supporting document. (PDF) [file pone.0305564.s001.pdf]

S1 Appendix Search Strategy

Embase ('gait stability'/de OR ((gait/de OR 'walking'/de) AND ('joint instability'/exp OR 'ankle instability'/de OR 'joint stability'/exp OR 'unsteadiness'/de OR 'balance disorder'/de)) OR ((gait OR walking OR bipedal) NEAR/3 (stabili* OR destabil* OR stable* OR instabil* OR unstable* OR unstead* OR symmetr* OR asymmetr* OR equilibri* OR balanc* OR imbalanc*)):ab,ti,kw) AND ('biomechanics'/de OR 'kinematics'/de OR 'gait kinematics'/de OR (biomechan* OR kinematic* OR perturb* OR ((influenc* OR augmentat* OR manipulat* OR assist*) NEAR/3 (stabilit* OR movement* OR gait* OR walk* OR ambulat* OR leg OR legs OR motion* OR balanc* OR postur*)) OR ((stabili* OR instabil* OR imbalanc*) NEAR/3 (environment* OR condition* OR force OR forces OR moment* OR torque*))):ab,ti,kw) AND ('devices'/de OR 'general device'/de OR 'medical device'/de OR 'orthosis'/exp OR 'rehabilitation equipment'/exp OR 'robotics'/de OR 'mechanics'/de OR 'force'/de OR 'torque'/de OR 'Performance Oriented Mobility Assessment'/de OR 'Berg Balance Scale'/de OR 'Dynamic Gait Index'/de OR (device* OR instrument* OR orthos* OR orthotic OR orthesis OR exoskelet* OR robot* OR tool* OR mechanic* OR stabiliz* OR stabilis* OR force OR forces OR haptic* OR moment* OR torque* OR Lyapunov* OR Floquet* OR variability-measure* OR long-range-correlation* OR extrapolated-centre*-of-mass OR xCOM* OR foot-placement-estimator* OR gait-sensitivit* OR Performance-Oriented-Mobility-Assess* OR POMA OR Tinetti OR Berg-Balance* OR BBS OR gait-index* OR margin*-of-stab* OR motoriz* OR ((walk* OR mobili* OR ambulat* OR motion*) NEAR/6 (aid* OR machine* OR equipment* OR system*)) OR walker* OR wearable*):ab,ti,kw) NOT ((animal/exp OR animal*:de OR nonhuman/de) NOT ('human'/exp)) AND [ENGLISH]/lim NOT ([Conference Abstract]/lim) NOT ('case report'/exp OR (case-report*):ti)

Medline (((Gait/ OR Walking/) AND (Joint Instability/ OR Postural Balance/)) OR ((gait OR walking OR bipedal) ADJ3 (stabili* OR destabil* OR stable* OR instabil* OR unstable* OR unstead* OR symmetr* OR asymmetr* OR equilibri* OR balanc* OR imbalanc*)).ab,ti,kf.) AND (Biomechanical Phenomena/ OR (biomechan* OR kinematic* OR perturb* OR ((influenc* OR augmentat* OR manipulat* OR assist*) ADJ3 (stabilit* OR movement* OR gait* OR walk* OR ambulat* OR leg OR legs OR motion* OR balanc* OR postur*)) OR ((stabili* OR instabil* OR imbalanc*) ADJ3 (environment* OR condition* OR force OR forces OR moment* OR torque*))).ab,ti,kf.) AND (Equipment and Supplies/ OR exp Orthotic Devices/ OR Canes/ OR Walkers/ OR Robotics/ OR Mechanics/ OR exp Torsion, Mechanical/ OR (device* OR instrument* OR orthos* OR orthotic OR orthesis OR exoskelet* OR robot* OR tool* OR mechanic* OR stabiliz* OR stabilis* OR force OR forces OR haptic* OR moment* OR torque* OR Lyapunov* OR Floquet* OR variability-measure* OR long-range-correlation* OR extrapolated-centre*-of-mass OR xCOM* OR foot-placement-estimator* OR gait-sensitivit* OR Performance-Oriented-Mobility-Assess* OR POMA OR Tinetti OR Berg-Balance* OR BBS OR gait-index*

OR margin*-of-stab* OR motoriz* OR ((walk* OR mobili* OR ambulat* OR motion*) ADJ6 (aid* OR machine* OR equipment* OR system*)) OR walker* OR wearable*).ab,ti,kf.) NOT (exp Animals/ NOT Humans/) AND english.la. NOT (news OR congres* OR abstract* OR book* OR chapter* OR dissertation abstract*).pt. NOT (Case Reports/ OR (case-report*).ti.)

Cochrane (((gait OR walking OR bipedal) NEAR/3 (stabili* OR destabil* OR stable* OR instabil* OR unstable* OR unstead* OR symmetr* OR asymmetr* OR equilibri* OR balanc* OR imbalanc*)):ab,ti) AND ((biomechan* OR kinematic* OR perturb* OR ((influenc* OR augmentat* OR manipulat* OR assist*) NEAR/3 (stabilit* OR movement* OR gait* OR walk* OR ambulat* OR leg OR legs OR motion* OR balanc* OR postur*)) OR ((stabili* OR instabil* OR imbalanc*) NEAR/3 (environment* OR condition* OR force OR forces OR moment* OR torque*))):ab,ti) AND ((device* OR instrument* OR orthos* OR orthotic OR orthesis OR exoskelet* OR robot* OR tool* OR mechanic* OR stabiliz* OR stabilis* OR force OR forces OR haptic* OR moment* OR torque* OR Lyapunov* OR Floquet* OR variability-measure* OR long-range-correlation* OR extrapolated-centre* NEXT of-mass OR xCOM* OR foot-placement-estimator* OR gait-sensitivit* OR Performance-Oriented-Mobility-Assess* OR POMA OR Tinetti OR Berg-Balance* OR BBS OR gait-index* OR margin* NEXT of-stab* OR motoriz* OR ((walk* OR mobili* OR ambulat* OR motion*) NEAR/6 (aid* OR machine* OR equipment* OR system*)) OR walker* OR wearable*):ab,ti)

Web of Science TS=((((gait OR walking OR bipedal) NEAR/2 (stabili* OR destabil* OR stable* OR instabil* OR unstable* OR unstead* OR symmetr* OR asymmetr* OR equilibri* OR balanc* OR imbalanc*))) AND ((biomechan* OR kinematic* OR perturb* OR ((influenc* OR augmentat* OR manipulat* OR assist*) NEAR/2 (stabilit* OR movement* OR gait* OR walk* OR ambulat* OR leg OR legs OR motion* OR balanc* OR postur*)) OR ((stabili* OR instabil* OR imbalanc*) NEAR/2 (environment* OR condition* OR force OR forces OR moment* OR torque*)))) AND ((device* OR instrument* OR orthos* OR orthotic OR orthesis OR exoskelet* OR robot* OR tool* OR mechanic* OR stabiliz* OR stabilis* OR force OR forces OR haptic* OR moment* OR torque* OR Lyapunov* OR Floquet* OR variability-measure* OR long-range-correlation* OR extrapolated-centre* NEXT of-mass OR xCOM* OR foot-placement-estimator* OR gait-sensitivit* OR Performance-Oriented-Mobility-Assess* OR POMA OR Tinetti OR Berg-Balance* OR BBS OR gait-index* OR margin* NEXT of-stab* OR motoriz* OR ((walk* OR mobili* OR ambulat* OR motion*) NEAR/5 (aid* OR machine* OR equipment* OR system*)) OR walker* OR wearable*)) NOT ((animal* OR rat OR rats OR mouse OR mice OR murine OR dog OR dogs OR canine OR cat OR cats OR feline OR rabbit OR cow OR cows OR bovine OR rodent* OR sheep OR ovine OR pig OR swine OR porcine OR veterinar* OR chick* OR zebrafish* OR baboon* OR nonhuman* OR primate* OR cattle* OR goose OR geese OR duck OR macaque* OR avian* OR bird* OR fish*) NOT (human* OR patient* OR

women OR woman OR men OR man))) AND DT=(Article OR Review) AND LA=(English) NOT TI=(case-report*)

Google Scholar ”gait | walking stability | instability | stable | unsteady | symmetry | asymmetry | equilibrium” augmentation | augmented | perturbation | perturbed device | instrument | orthosis | orthotic | orthesis | exoskeleton | robot | walker | ”walk | mobility aid | machine | equipment | system | tool”
