# Supplementary material for: Direct biomechanical manipulation of human gait stability: A systematic review
Source: PLoS One. 2024 Jul 11;19(7):e0305564. doi: 10.1371/journal.pone.0305564 (PMC11239080; doi:10.1371/journal.pone.0305564)
Supplement: S1 Table — The modified NIH quality and risk of bias assessment tool can be found here. (PDF) [file pone.0305564.s002.pdf]

S1 Table Quality and risk of bias assessment tool

Question	Score
1. Was the study question or objective clearly stated?	1
2. Were eligibility/selection criteria for the study population prespecified and clearly described?	1
3. Were the participants in the study representative of those who would be eligible for the test/service/intervention in the general or clinical population of interest? (0 – if device meant for patients, but only tested with non-impaired. 1 – if only non-impaired subjects used and device/study not intended for patients. 2 – if subjects are identical to intended patient population of device)	2
4. Were all eligible participants that met the prespecified entry criteria enrolled?	NA
5. Was the sample size sufficiently large to provide confidence in the findings? (1 - sample size is rationalized/defended. 2 – power calculation provided)	2
6. Was the test/service/intervention clearly described and delivered consistently across the study population?	1
7. Were the outcome measures prespecified, clearly defined, valid, reliable, and assessed consistently across all study participants?	1
8. Were the people assessing the outcomes blinded to the participants' exposures/interventions?	NA
9. Was the loss to follow-up after baseline 20% or less? Were those lost to follow-up accounted for in the analysis?	1
10. Did the statistical methods examine changes in outcome measures from before to during the intervention? Were statistical tests done that provided p values for the pre-to-post changes? (1 – if also included p-values, no correction for multiple measures. 2 – if also included p-values, plus correction for multiple measures)	2
11. Were outcome measures of interest taken multiple times before the intervention and multiple times during the intervention (i.e., did they use an interrupted time-series design)? (1 – if only done multiple times during the intervention. 2 – if also done multiple baseline measurements)	2
12. If the intervention was conducted at a group level (e.g., a whole hospital, a community, etc.) did the statistical analysis take into account the use of individual-level data to determine effects at the group level?	1
Total score	13
