# Supplementary material for: Direct biomechanical manipulation of human gait stability: A systematic review
Source: PLoS One. 2024 Jul 11;19(7):e0305564. doi: 10.1371/journal.pone.0305564 (PMC11239080; doi:10.1371/journal.pone.0305564)
Supplement: S2 Table — The full results of the quality and risk of bias assessment tool, for each article, can be found here. (PDF) [file pone.0305564.s003.pdf]

S2 Table Quality and risk of bias assessment scores

Author-Year	Ref.	Total	Q-1	Q-2	Q-3	Q-4	Q-5	Q-6	Q-7	Q-8	Q-9	Q-10	Q-11	Q-12
Zoffili-2017	[92]	9	1	0	1	-	0	1	1	-	1	2	2	-
Zhang-2018	[83]	10	1	1	2	-	0	1	1	-	1	1	2	-
Wan-2022	[86]	9	1	1	0	-	0	1	1	-	1	2	2	-
Sonntag-2000	[88]	10	1	1	2	-	0	1	0	-	1	2	2	-
Sorrento-2021	[64]	10	1	1	2	-	1	1	0	-	1	1	2	-
Seiferheld-2020	[85]	9	1	1	0	-	0	1	1	-	1	2	2	-
Polese-2012	[89]	11	1	1	2	-	2	1	1	-	1	2	0	-
Maguire-2010	[90]	9	1	1	2	-	0	1	1	-	1	2	0	-
Kloos-2012	[84]	11	1	1	2	-	0	1	1	-	1	2	2	-
Jayakaran-2014	[91]	9	1	1	0	-	1	0	1	-	1	2	2	-
Ijmker-2013a	[87]	13	1	1	2	-	2	1	1	-	1	2	2	-
Bannwart-2020	[61]	11	1	1	0	-	2	1	1	-	1	2	2	-
Best-2019	[72]	10	1	1	1	-	0	1	1	-	1	2	2	-
Clark-2012	[99]	9	1	1	2	-	2	1	0	-	0	2	0	-
Dragunas-2016	[70]	8	1	0	0	-	0	1	1	-	1	2	2	-
Goncalves-2017	[69]	6	0	1	0	-	0	1	1	-	1	0	2	-
Ignasiak-2019	[59]	9	1	1	1	-	0	0	1	-	1	2	2	-
Lemus-2020	[57]	8	1	0	0	-	0	1	1	-	1	2	2	-
Pillar-1991	[71]	6	1	0	2	-	0	1	0	-	1	1	0	-
Qian-2022	[68]	11	1	1	1	-	1	1	1	-	1	2	2	-
Livolsi-2022	[66]	11	1	1	2	-	0	1	1	-	1	2	2	-
Lee-2017	[54]	9	1	1	2	-	0	1	1	-	1	2	0	-
Monaco-2017	[63]	10	1	1	2	-	0	1	1	-	1	2	1	-
Park-2022	[96]	11	1	0	2	-	2	1	1	-	1	1	2	-
Hsu-2020	[55]	10	1	1	2	-	0	1	1	-	1	1	2	-
Norris-2007	[98]	8	1	0	0	-	0	1	1	-	1	2	2	-
Heitkamp-2019	[100]	9	1	0	1	-	0	1	1	-	1	2	2	-
Nyberg-2017	[101]	9	1	0	1	-	0	1	1	-	1	2	2	-
Reimold-2021	[102]	12	1	1	2	-	1	1	1	-	1	2	2	-
Yen-2015	[103]	11	1	1	2	-	0	1	1	-	1	2	2	-
Bacek-2022	[95]	11	1	1	1	-	1	1	1	-	1	2	2	-
Haufe-2021	[94]	10	1	1	1	-	0	1	1	-	1	2	2	-
Chinimilli-2020	[93]	8	1	0	0	-	0	1	1	-	1	2	2	-
Choi-2021	[67]	9	1	1	0	-	1	1	0	-	1	2	2	-
Galle-2017	[97]	10	1	1	2	-	0	1	1	-	1	1	2	-
Vashista-2013	[104]	9	1	0	1	-	0	1	1	-	1	2	2	-
Bruijn-2015	[58]	9	1	0	1	-	0	1	1	-	1	2	2	-
VanLeeuwen-2022	[79]	10	1	1	1	-	0	1	1	-	1	2	2	-
Dean-2007	[73]	11	1	1	2	-	0	1	1	-	1	2	2	-
Donelan-2004	[74]	10	1	1	1	-	0	1	1	-	1	2	2	-
Dragunas-2021	[80]	13	1	1	2	-	2	1	1	-	1	2	2	-
Frame-2020	[75]	11	1	0	2	-	2	1	1	-	0	2	2	-
Graham-2018	[105]	11	1	1	1	-	2	0	1	-	1	2	2	-
Ijmker-2013b	[76]	12	1	1	1	-	2	1	1	-	1	2	2	-
Koopman-2013	[77]	6	1	0	0	-	0	1	1	-	1	0	2	-
LinJ-2019a	[82]	13	1	1	2	-	2	1	1	-	1	2	2	-
LinJ-2019b	[62]	12	1	1	2	-	1	1	1	-	1	2	2	-
Mahaki-2019	[56]	11	1	1	1	-	2	1	1	-	1	1	2	-
Mahaki-2021	[16]	11	1	0	1	-	2	1	1	-	1	2	2	-
Matsubara-2015	[81]	11	1	1	2	-	0	1	1	-	1	2	2	-
Ochs-2021	[60]	11	1	1	2	-	0	1	1	-	1	2	2	-
Wu-2017	[78]	11	1	1	2	-	0	1	1	-	1	2	2	-
Walker-2018	[65]	10	1	1	1	-	0	1	1	-	1	2	2	-

References

[1] Berg WP, Alessio HM, Mills EM, Tong C. Correlates of Recurrent Falling in Independent Community-Dwelling Older Adults. Journal of Motor Behavior. 1997;29(1):5–16. doi:10.1080/00222899709603465.

[2] Bruijn SM, Meijer OG, Beek PJ, van Dieën JH. Assessing the stability of human locomotion: a review of current measures. Journal of The Royal Society Interface. 2013;10(83):20120999. doi:10.1098/rsif.2012.0999.

[3] Rubenstein LZ. Falls in older people: epidemiology, risk factors and strategies for prevention. Age and Ageing. 2006;35(suppl_2):ii37–ii41. doi:10.1093/ageing/afl084.

[4] Osoba MY, Rao AK, Agrawal SK, Lalwani AK. Balance and gait in the elderly: A contemporary review: Balance and Gait in the Elderly. Laryngoscope Investigative Otolaryngology. 2019;4(1):143–153. doi:10.1002/lio2.252.

[5] Schniepp R, Huppert A, Decker J, Schenkel F, Schlick C, Rasoul A, et al. Fall prediction in neurological gait disorders: differential contributions from clinical assessment, gait analysis, and daily-life mobility monitoring;268(9):3421–3434. doi:10.1007/s00415-021-10504-x.

[6] Pratt JE, Tedrake R. Velocity-Based Stability Margins for Fast Bipedal Walking. In: Diehl M, Mombaur K, editors. Fast Motions in Biomechanics and Robotics. vol. 340. Berlin, Heidelberg: Springer Berlin Heidelberg; 2006. p. 299–324. Available from: http://link.springer.com/10.1007/978-3-540-36119-0_14.

[7] Hof AL, Gazendam MGJ, Sinke WE. The condition for dynamic stability. Journal of Biomechanics. 2005;38(1):1–8. doi:10.1016/j.jbiomech.2004.03.025.

[8] Shirota C, van Asseldonk E, Matjačić Z, Vallery H, Barralon P, Maggioni S, et al. Robot-supported assessment of balance in standing and walking. Journal of NeuroEngineering and Rehabilitation. 2017;14(1):80. doi:10.1186/s12984-017-0273-7.

[9] England SA, Granata KP. The influence of gait speed on local dynamic stability of walking. Gait & posture. 2007;25(2):172–178. doi:10.1016/j.gaitpost.2006.03.003.

[10] Roeles S, Rowe PJ, Bruijn SM, Childs CR, Tarfali GD, Steenbrink F, et al. Gait stability in response to platform, belt, and sensory perturbations in young and older adults. Medical & Biological Engineering & Computing. 2018;56(12):2325–2335. doi:10.1007/s11517-018-1855-7.

[11] Mombaur K, Vallery H, Hu Y, Buchli J, Bhounsule P, Boaventura T, et al. Control of Motion and Compliance. Bioinspired Legged Locomotion. 2017; p. 135–346.

[12] Kuo AD, Donelan JM. Dynamic Principles of Gait and Their Clinical Implications. Physical Therapy. 2010;90(2):157–174. doi:10.2522/ptj.20090125.

[13] Neptune RR, Vistamehr A. Dynamic Balance During Human Movement: Measurement and Control Mechanisms. Journal of Biomechanical Engineering. 2019;141(7):070801. doi:10.1115/1.4042170.

[14] Hamacher D, Singh NB, Van Dieën JH, Heller MO, Taylor WR. Kinematic measures for assessing gait stability in elderly individuals: a systematic review. Journal of The Royal Society Interface. 2011;8(65):1682–1698. doi:10.1098/rsif.2011.0416.

[15] Mehdizadeh S. The largest Lyapunov exponent of gait in young and elderly individuals: A systematic review. Gait & Posture. 2018;60:241–250. doi:10.1016/j.gaitpost.2017.12.016.

[16] Mahaki M, T IJ, Houdijk H, Bruijn SM. How does external lateral stabilization constrain normal gait, apart from improving medio-lateral gait stability? R Soc open sci. 2021;8(3):202088. doi:10.1098/rsos.202088.

[17] Alizadehsaravi L, Bruijn SM, Muijres W, Koster RAJ, Van Dieën JH. Improvement in gait stability in older adults after ten sessions of standing balance training;17(7):e0242115. doi:10.1371/journal.pone.0242115.

[18] Matjačić Z, Zadravec M, Olenšek A. Feasibility of robot-based perturbed-balance training during treadmill walking in a high-functioning chronic stroke subject: a case-control study. Journal of NeuroEngineering and Rehabilitation. 2018;15(1):32. doi:10.1186/s12984-018-0373-z.

[19] McCrum C, Gerards MHG, Karamanidis K, Zijlstra W, Meijer K. A systematic review of gait perturbation paradigms for improving reactive stepping responses and falls risk among healthy older adults. European Review of Aging and Physical Activity. 2017;14(1):3. doi:10.1186/s11556-017-0173-7.

[20] Watson F, Fino PC, Thornton M, Heracleous C, Loureiro R, Leong JJH. Use of the margin of stability to quantify stability in pathologic gait – a qualitative systematic review. BMC Musculoskeletal Disorders. 2021;22(1):597. doi:10.1186/s12891-021-04466-4.

[21] Hamacher D, Liebl D, Hödl C, Heßler V, Kniewasser CK, Thönnessen T, et al. Gait Stability and Its Influencing Factors in Older Adults. Frontiers in Physiology. 2019;9:1955. doi:10.3389/fphys.2018.01955.

[22] Lee Y, Curuk E, Aruin AS. Effect of Light Finger Touch, a Cognitive Task, and Vision on Standing Balance in Stroke;53(2):157–165. doi:10.1080/00222895.2020.1742082.

[23] Sienko KH, Seidler RD, Carender WJ, Goodworth AD, Whitney SL, Peterka RJ. Potential Mechanisms of Sensory Augmentation Systems on Human Balance Control;9:944. doi:10.3389/fneur.2018.00944.

[24] Plooij M, Apte S, Keller U, Baines P, Sterke B, Asboth L, et al. Neglected physical human-robot interaction may explain variable outcomes in gait neurorehabilitation research. Science Robotics. 2021;6(58):eabf1888. doi:10.1126/scirobotics.abf1888.

[25] Baimyshev A, Finn-Henry M, Goldfarb M. Feasibility of a Wearable Cold-Gas Thruster for Fall Prevention;144(8):084501. doi:10.1115/1.4054529.

[26] Peters DM, O'Brien ES, Kamrud KE, Roberts SM, Rooney TA, Thibodeau KP, et al. Utilization of wearable technology to assess gait and mobility post-stroke: a systematic review. Journal of NeuroEngineering and Rehabilitation. 2021;18(1):67. doi:10.1186/s12984-021-00863-x.

[27] Slavin RE. Best-Evidence Synthesis: An Alternative to Meta-Analytic and Traditional Reviews. Educational Researcher. 1986;.

[28] Bramer WM, Rethlefsen ML, Mast F, Kleijnen J. Evaluation of a new method for librarian-mediated literature searches for systematic reviews. Research Synthesis Methods. 2018;9(4):510–520. doi:10.1002/jrsm.1279.

[29] Page MJ, McKenzie JE, Bossuyt PM, Boutron I, Hoffmann TC, Mulrow CD, et al. The PRISMA 2020 statement: an updated guideline for reporting systematic reviews. Systematic Reviews. 2021;10(1):89. doi:10.1186/s13643-021-01626-4.

[30] Middleton A, Fritz SL. Assessment of Gait, Balance, and Mobility in Older Adults: Considerations for Clinicians. Current Translational Geriatrics and Experimental Gerontology Reports. 2013;2(4):205–214. doi:10.1007/s13670-013-0057-2.

[31] Stokes HE, Thompson JD, Franz JR. The Neuromuscular Origins of Kinematic Variability during Perturbed Walking. Scientific Reports. 2017;7(1):808. doi:10.1038/s41598-017-00942-x.

[32] Vistamehr A, Kautz SA, Bowden MG, Neptune RR. Correlations between measures of dynamic balance in individuals with post-stroke hemiparesis. Journal of Biomechanics. 2016;49(3):396–400. doi:10.1016/j.jbiomech.2015.12.047.

[33] Nott CR, Neptune RR, Kautz SA. Relationships between frontal-plane angular momentum and clinical balance measures during post-stroke hemiparetic walking. Gait & Posture. 2014;39(1):129–134. doi:10.1016/j.gaitpost.2013.06.008.

[34] Bhat SG, Subramanian SC, Sugar TS, Redkar S. Application of Floquet Theory to Human Gait Kinematics and Dynamics. Journal of Mechanisms and Robotics. 2021;13(6):061003. doi:10.1115/1.4050199.

[35] Wodarski P, Jurkojć J, Polechoński J, Bieniek A, Chrzan M, Michnik R, et al. Assessment of gait stability and preferred walking speed in virtual reality. Acta of Bioengineering and Biomechanics. 2020;22(1). doi:10.37190/ABB-01490-2019-03.

[36] Riva F, Toebes MJP, Pijnappels M, Stagni R, van Dieën JH. Estimating fall risk with inertial sensors using gait stability measures that do not require step detection. Gait & Posture. 2013;38(2):170–174. doi:10.1016/j.gaitpost.2013.05.002.

[37] Hurt CP, Grabiner MD. Age-related differences in the maintenance of frontal plane dynamic stability while stepping to targets. Journal of Biomechanics. 2015;48(4):592–597. doi:10.1016/j.jbiomech.2015.01.003.

[38] Kao PC, Dingwell JB, Higginson JS, Binder-Macleod S. Dynamic instability during post-stroke hemiparetic walking. Gait & Posture. 2014;40(3):457–463. doi:10.1016/j.gaitpost.2014.05.014.

[39] Kim JH. Relationship between gait symmetry and functional balance, walking performance in subjects with stroke. The Journal of Korean Physical Therapy. 2014;26(1):1–8.

[40] An CM, Son YL, Park YH, Moon SJ. Relationship between dynamic balance and spatiotemporal gait symmetry in hemiplegic patients with chronic stroke. Hong Kong Physiotherapy Journal. 2017;37:19–24. doi:10.1016/j.hkpj.2017.01.002.

[41] Lockhart TE, Liu J. Differentiating fall-prone and healthy adults using local dynamic stability. Ergonomics. 2008;51(12):1860–1872. doi:10.1080/00140130802567079.

[42] Herman T, Giladi N, Gurevich T, Hausdorff JM. Gait instability and fractal dynamics of older adults with a "cautious" gait: why do certain older adults walk fearfully? Gait & Posture. 2005;21(2):178–185. doi:10.1016/j.gaitpost.2004.01.014.

[43] Pijnappels M, van der Burg PJCE, Reeves ND, van Dieën JH. Identification of elderly fallers by muscle strength measures. European Journal of Applied Physiology. 2008;102(5):585–592. doi:10.1007/s00421-007-0613-6.

[44] Dingwell JB. Lyapunov Exponents. In: Wiley Encyclopedia of Biomedical Engineering. John Wiley & Sons, Ltd; 2006.Available from: `https://onlinelibrary.wiley.com/doi/abs/10.1002/9780471740360.ebs0702`.

[45] Dingwell JB, Cusumano JP, Cavanagh PR, Sternad D. Local Dynamic Stability Versus Kinematic Variability of Continuous Overground and Treadmill Walking. Journal of Biomechanical Engineering. 2001;123(1):27–32. doi:10.1115/1.1336798.

[46] Herr H, Popovic M. Angular momentum in human walking. Journal of Experimental Biology. 2008;211(4):467–481. doi:10.1242/jeb.008573.

[47] Neptune RR, McGowan CP. Muscle contributions to frontal plane angular momentum during walking. Journal of Biomechanics. 2016;49(13):2975–2981. doi:10.1016/j.jbiomech.2016.07.016.

[48] Neptune RR, McGowan CP. Muscle contributions to whole-body sagittal plane angular momentum during walking. Journal of Biomechanics. 2011;44(1):6–12. doi:10.1016/j.jbiomech.2010.08.015.

[49] Hof AL. The 'extrapolated center of mass' concept suggests a simple control of balance in walking. Human Movement Science. 2008;27(1):112–125. doi:10.1016/j.humov.2007.08.003.

[50] Cromwell RL, Newton RA. Relationship between balance and gait stability in healthy older adults. Journal of Aging & Physical Activity. 2004;12(1).

[51] Balasubramanian CK, Neptune RR, Kautz SA. Variability in spatiotemporal step characteristics and its relationship to walking performance post-stroke. Gait & Posture. 2009;29(3):408–414. doi:10.1016/j.gaitpost.2008.10.061.

[52] Verlinden VJA, van der Geest JN, Hoogendam YY, Hofman A, Breteler MMB, Ikram MA. Gait patterns in a community-dwelling population aged 50 years and older. Gait & Posture. 2013;37(4):500–505. doi:10.1016/j.gaitpost.2012.09.005.

[53] National Heart aBIN Lung. Quality Assessment Tool for Before-After (Pre-Post) Studies With No Control Group; 2021. Available from: `https://www.nhlbi.nih.gov/health-topics/study-quality-assessment-tools`.

[54] Lee SH, Lee HJ, Chang WH, Choi BO, Lee J, Kim J, et al. Gait performance and foot pressure distribution during wearable robot-assisted gait in elderly adults. J NeuroEng Rehabil. 2017;14(1). doi:10.1186/s12984-017-0333-z.

[55] Hsu CC, Huang YK, Kang JH, Ko YF, Liu CW, Jaw FS, et al. Novel design for a dynamic ankle foot orthosis with motion feedback used for training in patients with hemiplegic gait: A pilot study. J NeuroEng Rehabil. 2020;17(1). doi:10.1186/s12984-020-00734-x.

[56] Mahaki M, Bruijn SM, Van Dieën JH. The effect of external lateral stabilization on the use of foot placement to control mediolateral stability in walking and running. PeerJ. 2019;2019(10). doi:10.7717/peerj.7939.

[57] Lemus D, Berry A, Jabeen S, Jayaraman C, Hohl K, van der Helm FCT, et al. Controller synthesis and clinical exploration of wearable gyroscopic actuators to support human balance. Sci rep. 2020;10(1):10412. doi:10.1038/s41598-020-66760-w.

[58] Bruijn SM, Van Dieën JH, Daffertshofer A. Beta activity in the premotor cortex is increased during stabilized as compared to normal walking. Front Human Neurosci. 2015;9(OCTOBER). doi:10.3389/fnhum.2015.00593.

[59] Ignasiak NK, Ravi DK, Orter S, Nasab SHH, Taylor WR, Singh NB. Does variability of footfall kinematics correlate with dynamic stability of the centre of mass during walking? PLoS ONE. 2019;14(5). doi:10.1371/journal.pone.0217460.

[60] Ochs WL, Woodward J, Cornwell T, Gordon KE. Meaningful measurements of maneuvers: People with incomplete spinal cord injury 'step up' to the challenges of altered stability requirements. J Neuroengineering Rehabil. 2021;18(1):46. doi:10.1186/s12984-021-00840-4.

[61] Bannwart M, Bayer SL, König Ignasiak N, Bolliger M, Rauter G, Easthope CA. Mediolateral damping of an overhead body weight support system assists stability during treadmill walking. J NeuroEng Rehabil. 2020;17(1). doi:10.1186/s12984-020-00735-w.

[62] Lin JT, Hsu CJ, Dee W, Chen D, Rymer WZ, Wu M. Error variability affects the after effects following motor learning of lateral balance control during walking in people with spinal cord injury. Eur J Neurosci. 2019;50(8):3221–3234. doi:10.1111/ejn.14478.

[63] Monaco V, Tropea P, Aprigliano F, Martelli D, Parri A, Cortese M, et al. An ecologically-controlled exoskeleton can improve balance recovery after slippage. Sci rep. 2017;7:46721.

[64] Sorrento GU, Archambault PS, Fung J. Walking with robot-generated haptic forces in a virtual environment: a new approach to analyze lower limb coordination. J NeuroEng Rehabil. 2021;18(1). doi:10.1186/s12984-021-00823-5.

[65] Walker ER, Hyngstrom AS, Onushko T, Schmit BD. Locomotor adaptations to prolonged step-by-step frontal plane trunk perturbations in young adults. PLoS ONE. 2018;13(9). doi:10.1371/journal.pone.0203776.

[66] Livolsi C, Conti R, Guanziroli E, Fririksson, Alexandersson A, Kristjansson K, et al. An impairment-specific hip exoskeleton assistance for gait training in subjects with acquired brain injury: a feasibility study. Sci rep. 2022;12(1):19343. doi:10.1038/s41598-022-23283-w.

[67] Choi HS, Baek YS, In H. Ankle strategy assistance to improve gait stability using controllers based on in-shoe center of pressure in 2 degree-of-freedom powered ankle-foot orthoses: a clinical study. J Neuroengineering Rehabil. 2021;19(1):114. doi:10.1186/s12984-022-01092-6.

[68] Qian Y, Yu H, Fu C. Adaptive Oscillator-Based Assistive Torque Control for Gait Asymmetry Correction With a nSEA-Driven Hip Exoskeleton. IEEE Trans Neural Syst Rehabil Eng. 2022;30:2906–2915. doi:10.1109/tnsre.2022.3213810.

[69] Goncalves RS, Krebs HI. MIT-Skywalker: considerations on the design of a body weight support system. J Neuroengineering Rehabil. 2017;14(1):88.

[70] Dragunas AC, Gordon KE. Body weight support impacts lateral stability during treadmill walking. Journal of Biomechanics. 2016;49(13):2662–2668. doi:10.1016/j.jbiomech.2016.05.026.

[71] Pillar T, Dickstein R, Smolinski Z. Walking reeducation with partial relief of body weight in rehabilitation of patients with locomotor disabilities. J REHABIL RES DEV. 1991;28(4):47–52.

[72] Best AN, Martin JP, Li Q, Wu AR. Stepping behaviour contributes little to balance control against continuous mediolateral trunk perturbations. J Exp Biol. 2019;222. doi:10.1242/jeb.212787.

[73] Dean JC, Alexander NB, Kuo AD. The effect of lateral stabilization on walking in young and old adults. IEEE Transactions on Biomedical Engineering. 2007;54(11):1919–1926. doi:10.1109/tbme.2007.901031.

[74] Donelan JM, Shipman DW, Kram R, Kuo AD. Mechanical and metabolic requirements for active lateral stabilization in human walking. Journal of Biomechanics. 2004;37(6):827–835. doi:10.1016/j.jbiomech.2003.06.002.

[75] Frame HB, Finetto C, Dean JC, Neptune RR. The influence of lateral stabilization on walking performance and balance control in neurologically-intact and post-stroke individuals. Clinical Biomechanics. 2020;73:172–180. doi:10.1016/j.clinbiomech.2020.01.005.

[76] Ijmker T, Houdijk H, Lamoth CJC, Beek PJ, van der Woude LHV. Energy cost of balance control during walking decreases with external stabilizer stiffness independent of walking speed. Journal of Biomechanics. 2013;46(13):2109–2114. doi:10.1016/j.jbiomech.2013.07.005.

[77] Koopman B, Meuleman JH, van Asseldonk EH, van der Kooij H. Lateral balance control for robotic gait training. IEEE Int Conf Rehabil Robot. 2013;2013:6650363. doi:10.1109/icorr.2013.6650363.

[78] Wu M, Brown G, Gordon KE. Control of locomotor stability in stabilizing and destabilizing environments. Gait and Posture. 2017;55:191–198. doi:10.1016/j.gaitpost.2017.04.021.

[79] van Leeuwen AM, van Dieen JH, Bruijn SM. The effect of external lateral stabilization on ankle moment control during steady-state walking. J Biomech. 2022;142:111259. doi:10.1016/j.jbiomech.2022.111259.

[80] Dragunas AC, Cornwell T, Lopez-Rosado R, Gordon KE. Post-Stroke Adaptation of Lateral Foot Placement Coordination in Variable Environments. IEEE Trans Neural Syst Rehabil Eng. 2021;29:731–739. doi:10.1109/tnsre.2021.3072252.

[81] Matsubara JH, Wu M, Gordon KE. Metabolic cost of lateral stabilization during walking in people with incomplete spinal cord injury. Gait and Posture. 2015;41(2):646–651. doi:10.1016/j.gaitpost.2015.01.015.

[82] Lin JT, Hsu CJ, Dee W, Chen D, Rymer WZ, Wu M. Motor Adaptation to Weight Shifting Assistance Transfers to Overground Walking in People with Spinal Cord Injury. Pm R. 2019;11(11):1200–1209. doi:10.1002/pmrj.12132.

[83] Zhang M, Artan NS, Gu H, Dong Z, Burina Ganatra L, Shermon S, et al. Gait study of parkinson's disease subjects using haptic cues with a motorized walker. Sensors (Basel). 2018;18(10). doi:10.3390/s18103549.

[84] Kloos AD, Kegelmeyer DA, White SE, Kostyk SK. The impact of different types of assistive devices on gait measures and safety in Huntington's disease. PLoS ONE. 2012;7(2). doi:10.1371/journal.pone.0030903.

[85] Seiferheld BE, Frost J, Andersen C, Samani A. New assistive walker improved local dynamic stability in young healthy adults. J Electromyogr Kinesiology. 2020;53. doi:10.1016/j.jelekin.2020.102441.

[86] Wan X, Yamada Y. An Acceleration-Based Nonlinear Time-Series Analysis of Effects of Robotic Walkers on Gait Dynamics During Assisted Walking. IEEE Sensors Journal. 2022;.

[87] Ijmker T, Houdijk H, Lamoth CJ, Jarbandhan AV, Rijntjes D, Beek PJ, et al. Effect of balance support on the energy cost of walking after stroke. Archives of Physical Medicine and Rehabilitation. 2013;94(11):2255–2261. doi:10.1016/j.apmr.2013.04.022.

[88] Sonntag D, Uhlenbrock D, Bardeleben A, Kading M, Hesse S. Gait with and without forearm crutches in patients with total hip arthroplasty. International Journal of Rehabilitation Research. 2000;23(3):233–243.

[89] Polese JC, Teixeira-Salmela LF, Nascimento LR, Faria CDM, Kirkwood RN, Laurentino GC, et al. The effects of walking sticks on gait kinematics and kinetics with chronic stroke survivors. Clinical Biomechanics. 2012;27(2):131–137. doi:10.1016/j.clinbiomech.2011.08.003.

[90] Maguire C, Sieben JM, Frank M, Romkes J. Hip abductor control in walking following stroke – the immediate effect of canes, taping and TheraTogs on gait. Clin Rehabil. 2010;24(1):37–45.

[91] Jayakaran P, DeSouza L, Cossar J, Gilhooly K. Influence of a walking aid on temporal and spatial parameters of gait in healthy adults. PM R. 2014;6(9):796–801. doi:10.1016/j.pmrj.2014.02.005.

[92] Zoffoli L, Ditroilo M, Federici A, Lucertini F. Local stability and kinematic variability in walking and pole walking at different speeds. Gait and Posture. 2017;53:1–4. doi:10.1016/j.gaitpost.2016.12.017.

[93] Chinimilli PT, Rezayat Sorkhabadi SM, Zhang W. Assessment of human dynamic gait stability with a lower extremity assistive device. IEEE transactions on neural systems and rehabilitation engineering : a publication of the IEEE Engineering in Medicine and Biology Society. 2020;28(3):669–678. doi:10.1109/tnsre.2020.2970207.

[94] Haufe FL, Kober AM, Wolf P, Riener R, Xiloyannis M. Learning to walk with a wearable robot in 880 simple steps: a pilot study on motor adaptation. J NeuroEng Rehabil. 2021;18(1). doi:10.1186/s12984-021-00946-9.

[95] Bacek T, Moltedo M, Serrien B, Langlois K, Vanderborght B, Lefeber D, et al. Human Musculoskeletal and Energetic Adaptations to Unilateral Robotic Knee Gait Assistance. IEEE Trans Biomed Eng. 2022;69(3):1141–1150. doi:10.1109/tbme.2021.3114737.

[96] Park JH, Kim S, Nussbaum MA, Srinivasan D. Effects of back-support exoskeleton use on gait performance and stability during level walking. Gait Posture. 2022;92:181–190. doi:10.1016/j.gaitpost.2021.11.028.

[97] Galle S, Derave W, Bossuyt F, Calders P, Malcolm P. Exoskeleton plantarflexion assistance for elderly. Gait & posture. 2017;.

[98] Norris JA, Marsh AP. Positive feedback in powered exoskeletons: Improved metabolic efficiency at the cost of reduced stability? 6th International Conference on Multibody Systems, Nonlinear Dynamics, and Control. 2007;.

[99] Clark RA, Williams G, Fini N, Moore L, Bryant AL. Coordination of dynamic balance during gait training in people with acquired brain injury. Archives of Physical Medicine and Rehabilitation. 2012;93(4):636–640. doi:10.1016/j.apmr.2011.11.002.

[100] Heitkamp LN, Stimpson KH, Dean JC. Application of a Novel Force-Field to Manipulate the Relationship between Pelvis Motion and Step Width in Human Walking. IEEE Trans Neural Syst Rehabil Eng. 2019;27(10):2051–2058. doi:10.1109/tnsre.2019.2941372.

[101] Nyberg ET, Broadway J, Finetto C, Dean JC. A Novel Elastic Force-Field to Influence Mediolateral Foot Placement during Walking. IEEE Trans Neural Syst Rehabil Eng. 2017;25(9):1481–1488. doi:10.1109/tnsre.2016.2633960.

[102] Reimold NK, Knapp HA, Chesnutt AN, Agne A, Dean JC. Effects of Targeted Assistance and Perturbations on the Relationship between Pelvis Motion and Step Width in People with Chronic Stroke. IEEE Trans Neural Syst Rehabil Eng. 2021;29:134–143. doi:10.1109/tnsre.2020.3038173.

[103] Yen SC, Schmit BD, Wu M. Using swing resistance and assistance to improve gait symmetry in individuals post-stroke. Human movement science. 2015;42:212–224. doi:10.1016/j.humov.2015.05.010.

[104] Vashista V, Agrawal N, Shaharudin S, Reisman DS, Agrawal SK. Force adaptation in human walking with symmetrically applied downward forces on the pelvis. Annu Int Conf IEEE Eng Med Biol Soc. 2013;2013:3312–3315. doi:10.1109/embc.2012.6346673.

[105] Graham SA, Hurt CP, Brown DA. Minimizing postural demands of walking while still emphasizing locomotor force generation for nonimpaired individuals. IEEE transactions on neural systems and rehabilitation engineering : a publication of the IEEE Engineering in Medicine and Biology Society. 2018;26(5):1003–1010.

[106] Bastian AJ. Understanding sensorimotor adaptation and learning for rehabilitation. Current Opinion in Neurology. 2008;21(6):628–633. doi:10.1097/WCO.0b013e328315a293.

[107] Poggensee KL, Collins SH. How adaptation, training, and customization contribute to benefits from exoskeleton assistance. SCIENCE ROBOTICS. 2021; p. 14.

[108] Son C, Lee A, Lee J, Kim D, Kim SJ, Chun MH, et al. The effect of pelvic movements of a gait training system for stroke patients: a single blind, randomized, parallel study. Journal of NeuroEngineering and Rehabilitation. 2021;18(1):185. doi:10.1186/s12984-021-00964-7.

[109] Apte S, Plooij M, Vallery H. Influence of body weight unloading on human gait characteristics: a systematic review. Journal of NeuroEngineering and Rehabilitation. 2018;15(1):53. doi:10.1186/s12984-018-0380-0.

[110] McAndrew Young PM, Dingwell JB. Voluntarily changing step length or step width affects dynamic stability of human walking. Gait & Posture. 2012;35(3):472–477. doi:10.1016/j.gaitpost.2011.11.010.

[111] Oates AR, Hauck L, Moraes R, Sibley KM. The effects of haptic input on biomechanical and neurophysiological parameters of walking: A scoping review. Gait & Posture. 2017;58:232–239. doi:10.1016/j.gaitpost.2017.08.004.

[112] Martins M, Santos C, Frizera A, Ceres R. A review of the functionalities of smart walkers. Medical Engineering & Physics. 2015;37(10):917–928. doi:10.1016/j.medengphy.2015.07.006.

[113] Mertens G, Krypotos AM. Preregistration of Analyses of Preexisting Data. Psychologica Belgica. 2019;59(1):338–352. doi:10.5334/pb.493.

[114] Sijtsma K, Emons WHM, Steneck NH, Bouter LM. Steps toward preregistration of research on research integrity. Research Integrity and Peer Review. 2021;6(1):5. doi:10.1186/s41073-021-00108-4.

[115] for Open Science C. The Open Science Framework; 2023. Available from: `https://osf.io/`.

[116] Geijtenbeek T. SCONE: Open Source Software for Predictive Simulation of Biological Motion;4(38):1421. doi:10.21105/joss.01421.

[117] Wyss D, Pennycott A, Bartenbach V, Riener R, Vallery H. A MUltidimensional Compliant Decoupled Actuator (MUCDA) for Pelvic Support During Gait. IEEE/ASME Transactions on Mechatronics. 2019;24(1):164–174. doi:10.1109/TMECH.2018.2878289.
